# Supplementary material for: Hybrid Boron-Carbon Chemistry
Source: Molecules. 2020 Oct 29;25(21):5026. doi: 10.3390/molecules25215026 (PMC7672580; doi:10.3390/molecules25215026)
Supplement: Supplementary file 1 [file molecules-25-05026-s001.pdf]

Hybrid Boron-Carbon Chemistry

Josep M. Oliva-Enrich [1,*], Ibon Alkorta [2] and José Elguero [2]

[1] Instituto de Química-Física "Rocasolano" (CSIC), Serrano 119, Madrid 28006, Spain; j.m.oliva@iqfr.csic.es

[2] Instituto de Quimica Médica (CSIC), Juan de la Cierva, 3, E-28006 Madrid, Spain; ibon@iqm.csic.es , iqmbe17@iqm.csic.es

* Correspondence: j.m.oliva@iqfr.csic.es , +34-915619400

Supplementary Information

B3LYP/cc-pVTZ optimised geometries, in cartesian coordinates (Å), of the systems included in the work.

Cyclobutadiene $C_{(4-2k)}B_{(2k)}H_{(4+2k)}$

C_4H_4 (k=0)

C	0.00000000	0.66449100	0.78749200
C	0.00000000	0.66449100	-0.78749200
C	0.00000000	-0.66449100	-0.78749200
C	0.00000000	-0.66449100	0.78749200
H	0.00000000	-1.42696700	1.55081200
H	0.00000000	-1.42696700	-1.55081200
H	0.00000000	1.42696700	-1.55081200
H	0.00000000	1.42696700	1.55081200

$C_2B_2H_6$ (k=1)

H	0.00000000	1.78498000	1.52627900
H	0.00000000	-1.78498000	1.52627900
H	0.00000000	-1.34894500	-1.66938200
H	0.00000000	1.34894500	-1.66938200
B	0.00000000	-0.87052800	0.77285400
B	0.00000000	0.87052800	0.77285400
H	-0.92909800	0.00000000	1.23502700
H	0.92909800	0.00000000	1.23502700
C	0.00000000	0.66904600	-0.82603200
C	0.00000000	-0.66904600	-0.82603200

B_4H_8 (k=2)

H	-1.71160800	1.71397900	0.00000000
H	-1.71160800	-1.71397900	0.00000000
H	1.71160800	-1.71397900	0.00000000
H	1.71160800	1.71397900	0.00000000
B	0.86381500	-0.87990900	0.00000000
B	0.86381500	0.87990900	0.00000000
B	-0.86381500	-0.87990900	0.00000000
B	-0.86381500	0.87990900	0.00000000
H	1.25257700	0.00000000	0.92784800
H	1.25257700	0.00000000	-0.92784800
H	-1.25257700	0.00000000	-0.92784800
H	-1.25257700	0.00000000	0.92784800

Benzene $C_{(6-2k)}B_{(2k)}H_{(6+2k)}$

C_6H_6 (k=0)

C	0.00000000	1.39076500	0.00000000
C	-1.20443800	0.69538200	0.00000000
C	-1.20443800	-0.69538200	0.00000000
C	0.00000000	-1.39076500	0.00000000
C	1.20443800	-0.69538200	0.00000000
C	1.20443800	0.69538200	0.00000000
H	0.00000000	2.47280200	0.00000000
H	-2.14150900	1.23640100	0.00000000
H	-2.14150900	-1.23640100	0.00000000
H	0.00000000	-2.47280200	0.00000000
H	2.14150900	-1.23640100	0.00000000
H	2.14150900	1.23640100	0.00000000

$C_4B_2H_8$ (k=1)

C	0.00000000	1.49120300	-0.10055000
C	0.00000000	-1.49120300	-0.10055000
C	0.00000000	-0.71930100	-1.21923400
C	0.00000000	0.71930100	-1.21923400
H	0.00000000	2.56640100	-0.24278400
H	0.00000000	1.54325200	2.31646900
H	0.00000000	-1.54325200	2.31646900
H	0.00000000	-2.56640100	-0.24278400
H	0.00000000	-1.19650700	-2.19420300
H	0.00000000	1.19650700	-2.19420300
B	0.00000000	-0.89939400	1.31987000
B	0.00000000	0.89939400	1.31987000
H	-0.96295600	0.00000000	1.43987600
H	0.96295600	0.00000000	1.43987600

$C_2B_4H_{10}$ (k=2)

B	0.00000000	1.60722500	-0.12028500
B	0.00000000	-1.60722500	-0.12028500
B	0.00000000	-0.84728100	1.49972800
B	0.00000000	0.84728100	1.49972800
H	0.00000000	-1.16961700	-2.34569700
H	0.00000000	-2.79016900	-0.23829300
H	0.00000000	1.54256800	2.46348600
H	0.00000000	2.79016900	-0.23829300
H	0.00000000	1.16961700	-2.34569700
H	0.00000000	-1.54256800	2.46348600
H	0.96318500	1.30844000	0.74133900
H	0.96318500	-1.30844000	0.74133900
H	-0.96318500	1.30844000	0.74133900
H	-0.96318500	-1.30844000	0.74133900
C	0.00000000	0.67520900	-1.37656400
C	0.00000000	-0.67520900	-1.37656400

B_6H_{12} (k=3)

B	-0.85625500	1.52943200	0.00000000
B	0.85625500	1.52943200	0.00000000
B	1.75265400	-0.02317800	0.00000000
B	0.89640000	-1.50625400	0.00000000
B	-0.89640000	-1.50625400	0.00000000
B	-1.75265400	-0.02317800	0.00000000
H	2.94137100	0.02759400	0.00000000
H	1.44678800	-2.56109900	0.00000000
H	-2.94137100	0.02759400	0.00000000
H	-1.49458300	2.53350500	0.00000000
H	1.49458300	2.53350500	0.00000000
H	-1.44678800	-2.56109900	0.00000000
H	-1.35280000	0.78103900	0.96131800
H	0.00000000	-1.56207900	0.96131800
H	1.35280000	0.78103900	0.96131800
H	-1.35280000	0.78103900	-0.96131800
H	0.00000000	-1.56207900	-0.96131800
H	1.35280000	0.78103900	-0.96131800

Cyclooctatetraene $C_{(8-2k)}B_{(2k)}H_{(8+2k)}$

C_8H_8 (k=0)

C	0.66759400	1.56391900	0.37091800
C	-0.66759400	1.56391900	0.37091800
C	-1.56391900	0.66759400	-0.37091800
C	1.56391900	0.66759400	-0.37091800
C	-1.56391900	-0.66759400	-0.37091800
C	1.56391900	-0.66759400	-0.37091800
C	-0.66759400	-1.56391900	0.37091800
C	0.66759400	-1.56391900	0.37091800
H	-1.16679300	2.35821000	0.91903900
H	1.16679300	2.35821000	0.91903900
H	2.35821000	1.16679300	-0.91903900
H	2.35821000	-1.16679300	-0.91903900
H	1.16679300	-2.35821000	0.91903900
H	-1.16679300	-2.35821000	0.91903900
H	-2.35821000	-1.16679300	-0.91903900
H	-2.35821000	1.16679300	-0.91903900

$C_6B_2H_{10}$ (k=1)

C	-0.71054900	0.22540200	1.70127100
C	-0.71054900	0.22540200	-1.70127100
C	0.14155700	-0.80183400	1.60497000
C	0.14155700	-0.80183400	-1.60497000
C	1.26135300	-0.98030400	0.66963000
C	1.26135300	-0.98030400	-0.66963000
H	-0.73412500	2.60664200	-1.44709900
H	-1.43044400	0.16555000	-2.51463000
H	0.07450600	-1.59351300	-2.34913200
H	2.20034800	-1.24373700	-1.15006700
H	2.20034800	-1.24373700	1.15006700
H	0.07450600	-1.59351300	2.34913200
H	-1.43044400	0.16555000	2.51463000
B	-0.71054900	1.55711000	-0.88501000
B	-0.71054900	1.55711000	0.88501000
H	0.26039900	1.61318600	0.00000000
H	-1.68380300	1.62665700	0.00000000
H	-0.73412500	2.60664200	1.44709900

$C_4B_4H_{12}$ (k=2) Isomer I

C	-1.78272300	0.67072000	-0.40761300
C	1.78272300	0.67072000	-0.40761300
C	-1.78272300	-0.67072000	-0.40761300
C	1.78272300	-0.67072000	-0.40761300
H	1.40346300	2.53506300	1.03984000
H	2.56280200	1.15373100	-0.99685100
H	2.56280200	-1.15373100	-0.99685100
H	1.40346300	-2.53506300	1.03984000
H	-1.40346300	-2.53506300	1.03984000
H	-2.56280200	-1.15373100	-0.99685100
H	-2.56280200	1.15373100	-0.99685100
B	0.89249700	1.67224800	0.39857400
B	-0.89249700	1.67224800	0.39857400
H	0.00000000	1.10144800	1.17997200
H	0.00000000	2.28118300	-0.36033300
H	-1.40346300	2.53506300	1.03984000
B	0.89249700	-1.67224800	0.39857400
B	-0.89249700	-1.67224800	0.39857400
H	0.00000000	-2.28118300	-0.36033300
H	0.00000000	-1.10144800	1.17997200

$C_4B_4H_{12}$ (k=2) Isomer II

C	-0.95783000	1.37512200	-0.75133900
C	-0.59567000	0.43447500	-1.63469300
H	0.05706800	3.04401900	0.61174900
H	-1.84988200	1.93896000	-1.01848300
H	-1.19603600	0.33360300	-2.53774200
H	1.19603600	-0.33360300	-2.53774200
H	1.84988200	-1.93896000	-1.01848300
H	-0.05706800	-3.04401900	0.61174900
H	-0.78903300	-1.32404500	2.79004200
B	-0.20727800	1.88614800	0.52187200
B	0.20727800	0.82135900	1.88100200
H	0.86386300	1.18116500	0.80001800
H	-0.83349200	1.57429700	1.65623800
H	0.78903300	1.32404500	2.79004200
B	-0.20727800	-0.82135900	1.88100200
B	0.20727800	-1.88614800	0.52187200
H	-0.86386300	-1.18116500	0.80001800
H	0.83349200	-1.57429700	1.65623800
C	0.59567000	-0.43447500	-1.63469300
C	0.95783000	-1.37512200	-0.75133900

$C_2B_6H_{14}$ (k=3)

C	0.46566300	-1.72870900	0.67162800
C	0.46566300	-1.72870900	-0.67162800
H	-1.10272600	-1.57125800	2.45972900
H	1.13708300	-2.44396000	1.14894800
H	1.13708300	-2.44396000	-1.14894800
H	-1.10272600	-1.57125800	-2.45972900
H	-1.22717200	1.24578400	-2.66109700
H	1.17745600	2.66404100	-1.41750900
H	1.17745600	2.66404100	1.41750900
B	-0.44068300	-0.95340900	1.68626800
B	-0.48434900	0.82652000	1.83051900
H	-1.20064700	-0.03314800	1.13771200
H	0.25057600	-0.07631300	2.41970800
H	-1.22717200	1.24578400	2.66109700
B	-0.44068300	-0.95340900	-1.68626800
B	-0.48434900	0.82652000	-1.83051900
H	0.25057600	-0.07631300	-2.41970800
H	-1.20064700	-0.03314800	-1.13771200
B	0.46566300	1.86874500	0.88828500
B	0.46566300	1.86874500	-0.88828500
H	1.18831300	1.22906100	0.00000000
H	-0.25172400	2.52658400	0.00000000

B_8H_{16} (k=4)

H	-2.63303000	-1.40454800	1.25187700
H	-2.63303000	1.40454800	1.25187700
H	-1.40454800	2.63303000	-1.25187700
H	1.40454800	2.63303000	-1.25187700
H	2.63303000	1.40454800	1.25187700
H	2.63303000	-1.40454800	1.25187700
H	1.40454800	-2.63303000	-1.25187700
H	-1.40454800	-2.63303000	-1.25187700
B	-0.89295500	-1.86392500	-0.49963500
B	-1.86392500	0.89295500	0.49963500
B	-1.86392500	-0.89295500	0.49963500
B	-0.89295500	1.86392500	-0.49963500
B	0.89295500	-1.86392500	-0.49963500
B	0.89295500	1.86392500	-0.49963500
B	1.86392500	-0.89295500	0.49963500
B	1.86392500	0.89295500	0.49963500
H	-1.19454700	0.00000000	1.19208500
H	0.00000000	2.53825200	0.19902400
H	1.19454700	0.00000000	1.19208500
H	0.00000000	-2.53825200	0.19902400
H	-2.53825200	0.00000000	-0.19902400
H	0.00000000	-1.19454700	-1.19208500
H	2.53825200	0.00000000	-0.19902400
H	0.00000000	1.19454700	-1.19208500

Benzocyclobutadiene Kekulé Structure K_1 : $C_{(8-2k)}B_{(2k)}H_{(6+2k)}$

C_8H_6 (k=0) - same energy for Kekulé structures K_1, K_2 and K_3

C	0.00000000	0.68599500	-1.83961000
C	0.00000000	1.43946700	-0.62161400
C	0.00000000	0.70954000	0.52046900
C	0.00000000	-0.70954000	0.52046900
C	0.00000000	-1.43946700	-0.62161400
C	0.00000000	-0.68599500	-1.83961000
H	0.00000000	1.21700600	-2.78214600
H	0.00000000	2.52090300	-0.64319100
C	0.00000000	0.67295800	2.04188100
C	0.00000000	-0.67295800	2.04188100
H	0.00000000	-2.52090300	-0.64319100
H	0.00000000	-1.21700600	-2.78214600
H	0.00000000	-1.42183200	2.81858700
H	0.00000000	1.42183200	2.81858700

$C_6B_2H_8$ (k=1) Isomer I

H	0.00000000	-1.22482500	-2.82319600
H	0.00000000	-2.49922800	-0.69308100
H	0.00000000	2.49922800	-0.69308100
H	0.00000000	1.22482500	-2.82319600
H	0.00000000	1.78527500	2.85974600
H	0.00000000	-1.78527500	2.85974600
B	0.00000000	-0.88719300	2.08761500
B	0.00000000	0.88719300	2.08761500
H	-0.93056700	0.00000000	2.51413900
H	0.93056700	0.00000000	2.51413900
C	0.00000000	-0.69263200	-1.88068500
C	0.00000000	-1.41627600	-0.67422400
C	0.00000000	-0.70716500	0.50562800
C	0.00000000	0.70716500	0.50562800
C	0.00000000	0.69263200	-1.88068500
C	0.00000000	1.41627600	-0.67422400

$C_6B_2H_8$ (k=1) Isomer II

H	0.00000000	-1.16780600	-2.85294600
H	0.00000000	-2.63472900	-0.97016700
H	0.00000000	2.63472900	-0.97016700
H	0.00000000	1.16780600	-2.85294600
H	0.00000000	1.32926600	3.07776000
H	0.00000000	-1.32926600	3.07776000
B	0.00000000	-0.87467500	0.59557200
B	0.00000000	0.87467500	0.59557200
H	-0.94191100	0.00000000	0.29877200
H	0.94191100	0.00000000	0.29877200
C	0.00000000	-0.67382400	2.21624300
C	0.00000000	0.67382400	2.21624300
C	0.00000000	-1.56874000	-0.77407700
C	0.00000000	-0.72097800	-1.86404600
C	0.00000000	0.72097800	-1.86404600
C	0.00000000	1.56874000	-0.77407700

$C_6B_2H_8$ (k=1) Isomer III

H	3.06512000	-0.57981400	0.00000000
H	2.00847500	1.59334400	0.00000000
H	-1.38971300	-2.56271300	0.00000000
H	1.68366000	-2.94252800	0.00000000
H	-3.11956800	0.92020700	0.00000000
H	-1.14682400	2.98085000	0.00000000
B	1.18913700	-1.86404800	0.00000000
B	-0.63574000	-1.64808800	0.00000000
H	0.27628400	-1.84702600	0.96092000
H	0.27628400	-1.84702600	-0.96092000
C	0.00000000	0.82659100	0.00000000
C	-1.11960100	1.90192200	0.00000000
C	-2.04034100	0.93355900	0.00000000
C	-0.95326900	-0.16867100	0.00000000
C	1.98077100	-0.55058200	0.00000000
C	1.39565500	0.69807800	0.00000000

$C_4B_4H_{10}$ (k=2) Isomer I

H	3.09875100	-0.58345800	0.00000000
H	2.02925800	1.56039300	0.00000000
H	-1.32597900	-2.58722100	0.00000000
H	1.73847600	-2.96097500	0.00000000
H	-3.39005000	0.65314500	0.00000000
H	-0.94482300	3.25628600	0.00000000
B	1.22666800	-1.88971600	0.00000000
B	-0.57467500	-1.66809700	0.00000000
B	-1.02568300	2.07364800	0.00000000
B	-2.21282200	0.78269000	0.00000000
H	-1.92940500	1.74774100	0.93067000
H	-1.92940500	1.74774100	-0.93067000
H	0.32244800	-1.89127600	0.96189100
H	0.32244800	-1.89127600	-0.96189100
C	2.01375300	-0.56401100	0.00000000
C	1.41548800	0.66435400	0.00000000
C	0.00000000	0.82653200	0.00000000
C	-0.93910200	-0.18416200	0.00000000

$C_4B_4H_{10}$ (k=2) Isomer II

H	0.00000000	1.17118600	-2.91266800
H	0.00000000	2.62285400	-1.02418100
H	0.00000000	-2.62285400	-1.02418100
H	0.00000000	-1.17118600	-2.91266800
H	0.00000000	-1.70021600	3.17603800
H	0.00000000	1.70021600	3.17603800
B	0.00000000	0.89615700	0.55936200
B	0.00000000	-0.89615700	0.55936200
B	0.00000000	0.89716100	2.29973000
B	0.00000000	-0.89716100	2.29973000
H	0.92833400	0.00000000	2.66537700
H	-0.92833400	0.00000000	2.66537700
H	0.93933200	0.00000000	0.34505900
H	-0.93933200	0.00000000	0.34505900
C	0.00000000	1.55536300	-0.83316300
C	0.00000000	0.72173500	-1.92435100
C	0.00000000	-0.72173500	-1.92435100
C	0.00000000	-1.55536300	-0.83316300

$C_4B_4H_{10}$ (k=2) Isomer III

H	3.13307200	-0.51325900	0.00000000
H	2.24367400	1.66982600	0.00000000
H	-1.05517700	-2.98209400	0.00000000
H	1.93169200	-2.81746400	0.00000000
H	-3.28991700	0.98362000	0.00000000
H	-1.53646200	2.99747700	0.00000000
B	1.29112800	-1.81585000	0.00000000
B	-0.53711800	-1.91451700	0.00000000
B	0.00000000	1.01897900	0.00000000
B	-1.13217100	-0.32442800	0.00000000
H	-0.33398100	0.09340900	0.93354800
H	0.39394300	-1.97024900	0.95706800
H	-0.33398100	0.09340900	-0.93354800
H	0.39394300	-1.97024900	-0.95706800
C	-1.32780500	1.93502200	0.00000000
C	-2.20831500	0.91939000	0.00000000
C	1.54519600	0.83802100	0.00000000
C	2.04825800	-0.42665800	0.00000000

$C_4B_4H_{10}$ (k=2) Isomer IV

H	0.00000000	1.53366700	-3.14216600
H	0.00000000	2.83761900	-0.44271200
H	0.00000000	-2.83761900	-0.44271200
H	0.00000000	-1.53366700	-3.14216600
H	0.00000000	-1.42802600	2.97066400
H	0.00000000	1.42802600	2.97066400
B	0.00000000	0.84632000	-2.17251400
B	0.00000000	-0.84632000	-2.17251400
B	0.00000000	1.65543400	-0.55446700
B	0.00000000	-1.65543400	-0.55446700
H	0.96249800	1.32109200	-1.41506800
H	0.96249800	-1.32109200	-1.41506800
H	-0.96249800	1.32109200	-1.41506800
H	-0.96249800	-1.32109200	-1.41506800
C	0.00000000	0.68147900	0.64137500
C	0.00000000	0.66621700	2.20516800
C	0.00000000	-0.66621700	2.20516800
C	0.00000000	-0.68147900	0.64137500

$C_2B_6H_{12}$ (k=3) Isomer I

0 1

H	0.00000000	1.53475500	-3.18619800
H	0.00000000	2.82092800	-0.49729900
H	0.00000000	-2.82092800	-0.49729900
H	0.00000000	-1.53475500	-3.18619800
H	0.00000000	-1.78308600	2.99683100
H	0.00000000	1.78308600	2.99683100
B	0.00000000	0.84841600	-2.21529600
B	0.00000000	-0.84841600	-2.21529600
B	0.00000000	1.63605700	-0.59800500
B	0.00000000	-1.63605700	-0.59800500
B	0.00000000	0.87233600	2.23774800
B	0.00000000	-0.87233600	2.23774800
H	0.92940400	0.00000000	2.68707900
H	-0.92940400	0.00000000	2.68707900
H	0.96219600	1.31897400	-1.46112900
H	0.96219600	-1.31897400	-1.46112900
H	-0.96219600	1.31897400	-1.46112900
H	-0.96219600	-1.31897400	-1.46112900
C	0.00000000	0.68461600	0.63326800
C	0.00000000	-0.68461600	0.63326800

$C_2B_6H_{12}$ (k=3) Isomer II

H	3.20683500	-0.42527500	0.00000000
H	2.21677100	1.70854600	0.00000000
H	-0.87874400	-3.03617000	0.00000000
H	2.08867100	-2.78049700	0.00000000
H	-3.63700100	0.60480600	0.00000000
H	-1.52678900	3.27855100	0.00000000
B	1.41621200	-1.79940400	0.00000000
B	-0.39748400	-1.95046800	0.00000000
B	0.00000000	1.01374500	0.00000000
B	-1.10126000	-0.40153300	0.00000000
B	-1.35429800	2.10158800	0.00000000
B	-2.45161000	0.69765000	0.00000000
H	-2.19192400	1.63782400	0.92792000
H	-2.19192400	1.63782400	-0.92792000
H	0.52072200	-1.97202000	0.95824900
H	-0.36160100	0.12776000	0.93190500
H	0.52072200	-1.97202000	-0.95824900
H	-0.36160100	0.12776000	-0.93190500
C	2.11892000	-0.38777700	0.00000000
C	1.55409000	0.84694700	0.00000000

$C_2B_6H_{12}$ (k=3) Isomer III

H	0.00000000	1.45299800	-3.22415100
H	0.00000000	3.04861400	-0.76083300
H	0.00000000	-3.04861400	-0.76083300
H	0.00000000	-1.45299800	-3.22415100
H	0.00000000	-1.33889700	3.19630700
H	0.00000000	1.33889700	3.19630700
B	0.00000000	0.86596200	-2.18830700
B	0.00000000	-0.86596200	-2.18830700
B	0.00000000	1.86415000	-0.67200600
B	0.00000000	-1.86415000	-0.67200600
B	0.00000000	0.88214000	0.73003100
B	0.00000000	-0.88214000	0.73003100
H	0.95776200	1.42935700	-1.48555700
H	0.95776200	-1.42935700	-1.48555700
H	0.92216200	0.00000000	0.35254100
H	-0.95776200	1.42935700	-1.48555700
H	-0.95776200	-1.42935700	-1.48555700
H	-0.92216200	0.00000000	0.35254100
C	0.00000000	0.67085600	2.34311000
C	0.00000000	-0.67085600	2.34311000

B_8H_{14} (k=4) Kekulé Structure K_1

H	0.00000000	1.46161700	-3.29102100
H	0.00000000	3.02425400	-0.82631000
H	0.00000000	-3.02425400	-0.82631000
H	0.00000000	-1.46161700	-3.29102100
H	0.00000000	-1.70480500	3.30236000
H	0.00000000	1.70480500	3.30236000
B	0.00000000	0.86328300	-2.26137300
B	0.00000000	-0.86328300	-2.26137300
B	0.00000000	1.83875300	-0.73701000
B	0.00000000	-1.83875300	-0.73701000
B	0.00000000	0.89906900	0.69957100
B	0.00000000	-0.89906900	0.69957100
B	0.00000000	0.88755600	2.43746000
B	0.00000000	-0.88755600	2.43746000
H	0.92784800	0.00000000	2.81508300
H	-0.92784800	0.00000000	2.81508300
H	0.92264200	0.00000000	0.40034300
H	0.95824100	1.40859900	-1.54684600
H	0.95824100	-1.40859900	-1.54684600
H	-0.92264200	0.00000000	0.40034300
H	-0.95824100	1.40859900	-1.54684600
H	-0.95824100	-1.40859900	-1.54684600

Benzocyclobutadiene Kekulé Structure K_2 : $C_{(8-2k)}B_{(2k)}H_{(6+2k)}$

$C_6B_2H_8$ (k=1) Isomer I

H	0.00000000	1.22474200	-2.82314900
H	0.00000000	2.49910000	-0.69295500
H	0.00000000	-2.49910000	-0.69295500
H	0.00000000	-1.22474200	-2.82314900
H	0.00000000	-1.78549500	2.85909500
H	0.00000000	1.78549500	2.85909500
B	0.00000000	0.88688500	2.08756600
B	0.00000000	-0.88688500	2.08756600
H	0.93045400	0.00000000	2.51405600
H	-0.93045400	0.00000000	2.51405600
C	0.00000000	1.41621100	-0.67424100
C	0.00000000	0.70728000	0.50576000
C	0.00000000	-0.70728000	0.50576000
C	0.00000000	-1.41621100	-0.67424100
C	0.00000000	0.69267800	-1.88066600
C	0.00000000	-0.69267800	-1.88066600

$C_6B_2H_8$ (k=1) Isomer II

H	0.00000000	1.52254600	-2.95057100
H	0.00000000	2.63321900	-0.38473200
H	0.00000000	-2.63321900	-0.38473200
H	0.00000000	-1.52254600	-2.95057100
H	0.00000000	-1.42294500	2.81287300
H	0.00000000	1.42294500	2.81287300
B	0.00000000	0.90189700	-1.93844800
B	0.00000000	-0.90189700	-1.93844800
H	0.96276900	0.00000000	-2.07417400
H	-0.96276900	0.00000000	-2.07417400
C	0.00000000	0.67637600	2.03292400
C	0.00000000	-0.67637600	2.03292400
C	0.00000000	-0.73639600	0.53661500
C	0.00000000	-1.55778600	-0.52139900
C	0.00000000	1.55778600	-0.52139900
C	0.00000000	0.73639600	0.53661500

$C_6B_2H_8$ (k=1) Isomer III

H	1.80859200	2.62397200	0.00000000
H	-0.90033700	2.89135500	0.00000000
H	2.00767600	-1.64411500	0.00000000
H	3.00531400	0.59334500	0.00000000
H	-1.16759600	-2.92692400	0.00000000
H	-3.20968000	-1.08688700	0.00000000
B	-0.33141500	1.84883200	0.00000000
B	-1.05009200	0.24132300	0.00000000
H	-0.92759700	1.13198300	0.96355700
H	-0.92759700	1.13198300	-0.96355700
C	-2.14111600	-0.92870700	0.00000000
C	-1.13791300	-1.84095900	0.00000000
C	0.00000000	-0.89228500	0.00000000
C	1.33837400	-0.79034100	0.00000000
C	1.22327800	1.71101000	0.00000000
C	1.92050300	0.54703400	0.00000000

$C_4B_4H_{10}$ (k=2) Isomer I

H	0.00000000	1.52856500	-3.00313300
H	0.00000000	2.60398800	-0.44239600
H	0.00000000	-2.60398800	-0.44239600
H	0.00000000	-1.52856500	-3.00313300
H	0.00000000	-1.78587100	2.87516400
H	0.00000000	1.78587100	2.87516400
B	0.00000000	0.90123000	-1.99547900
B	0.00000000	-0.90123000	-1.99547900
B	0.00000000	0.89119200	2.09949700
B	0.00000000	-0.89119200	2.09949700
H	0.96318700	0.00000000	-2.11238700
H	0.93146600	0.00000000	2.50137000
H	-0.96318700	0.00000000	-2.11238700
H	-0.93146600	0.00000000	2.50137000
C	0.00000000	1.52628800	-0.57583000
C	0.00000000	0.73496900	0.51937900
C	0.00000000	-0.73496900	0.51937900
C	0.00000000	-1.52628800	-0.57583000

$C_4B_4H_{10}$ (k=2) Isomer II

H	-1.91475000	-2.62374500	0.00000000
H	0.78896100	-2.92075100	0.00000000
H	-2.00039700	1.62770600	0.00000000
H	-3.06143600	-0.55092200	0.00000000
H	1.06368600	3.21193100	0.00000000
H	3.56825800	0.78432600	0.00000000
B	0.23491300	-1.86975100	0.00000000
B	1.02018800	-0.28489800	0.00000000
B	2.38218300	0.76830200	0.00000000
B	1.08610500	2.02270900	0.00000000
H	0.84034400	-1.15370200	0.96139900
H	2.00948600	1.64488000	0.93094400
H	0.84034400	-1.15370200	-0.96139900
H	2.00948600	1.64488000	-0.93094400
C	-1.30793100	-1.72447400	0.00000000
C	-1.97570700	-0.54181500	0.00000000
C	-1.34318300	0.76176600	0.00000000
C	0.00000000	0.88907200	0.00000000

$C_4B_4H_{10}$ (k=2) Isomer III

H	1.85299400	2.94940800	0.00000000
H	-1.20888700	2.86905200	0.00000000
H	1.86835300	-1.91209600	0.00000000
H	3.32030200	0.42633500	0.00000000
H	-1.22414200	-2.91485500	0.00000000
H	-3.22156600	-1.02795700	0.00000000
B	1.21936500	1.94268400	0.00000000
B	2.13259400	0.38275000	0.00000000
B	-0.49969100	1.91400000	0.00000000
B	-1.05158200	0.24777700	0.00000000
H	-0.98804500	1.16507100	0.96271000
H	1.71803600	1.23270700	0.96382800
H	-0.98804500	1.16507100	-0.96271000
H	1.71803600	1.23270700	-0.96382800
C	-2.14981800	-0.89131600	0.00000000
C	-1.16671800	-1.82940800	0.00000000
C	0.00000000	-0.92158500	0.00000000
C	1.34145900	-0.96127500	0.00000000

$C_4B_4H_{10}$ (k=2) Isomer IV

H	0.00000000	1.15125100	-3.02622800
H	0.00000000	2.85462700	-1.00520300
H	0.00000000	-2.85462700	-1.00520300
H	0.00000000	-1.15125100	-3.02622800
H	0.00000000	-1.33319800	3.14123600
H	0.00000000	1.33319800	3.14123600
B	0.00000000	1.67741100	-0.82175500
B	0.00000000	-1.67741100	-0.82175500
B	0.00000000	0.83247700	0.70237500
B	0.00000000	-0.83247700	0.70237500
H	0.95981300	1.49089300	0.06969300
H	0.95981300	-1.49089300	0.06969300
H	-0.95981300	1.49089300	0.06969300
H	-0.95981300	-1.49089300	0.06969300
C	0.00000000	0.67987800	2.27240300
C	0.00000000	-0.67987800	2.27240300
C	0.00000000	0.67410400	-2.04778400
C	0.00000000	-0.67410400	-2.04778400

$C_2B_6H_{12}$ (k=3) Isomer I

H	-3.37230500	0.40083800	0.00000000
H	-1.87807200	-1.86828800	0.00000000
H	1.10471600	2.89145200	0.00000000
H	-1.95717300	2.97047600	0.00000000
H	3.57927500	-0.75633200	0.00000000
H	1.11301600	-3.22179100	0.00000000
B	-2.18372400	0.40669200	0.00000000
B	-1.30687400	1.97515200	0.00000000
B	0.39787700	1.93414200	0.00000000
B	1.02459300	0.28476700	0.00000000
B	1.11764000	-2.03249600	0.00000000
B	2.39321300	-0.75671200	0.00000000
H	-1.79669600	1.24349500	0.96363000
H	0.90594200	1.17612800	0.95932300
H	2.02681800	-1.63426700	0.93142900
H	-1.79669600	1.24349500	-0.96363000
H	0.90594200	1.17612800	-0.95932300
H	2.02681800	-1.63426700	-0.93142900
C	-1.34586900	-0.91760000	0.00000000
C	0.00000000	-0.92319800	0.00000000

$C_2B_6H_{12}$ (k=3) Isomer II

H	0.00000000	1.15926300	-3.09763100
H	0.00000000	2.82063900	-1.05248800
H	0.00000000	-2.82063900	-1.05248800
H	0.00000000	-1.15926300	-3.09763100
H	0.00000000	-1.69913200	3.27081600
H	0.00000000	1.69913200	3.27081600
B	0.00000000	1.64080500	-0.88315000
B	0.00000000	-1.64080500	-0.88315000
B	0.00000000	0.84661900	0.67092500
B	0.00000000	-0.84661900	0.67092500
B	0.00000000	0.90984200	2.37926400
B	0.00000000	-0.90984200	2.37926400
H	0.95798700	1.45576900	0.04157100
H	0.95798700	-1.45576900	0.04157100
H	0.93175400	0.00000000	2.70143800
H	-0.95798700	1.45576900	0.04157100
H	-0.95798700	-1.45576900	0.04157100
H	-0.93175400	0.00000000	2.70143800
C	0.00000000	0.67471900	-2.12341200
C	0.00000000	-0.67471900	-2.12341200

$C_2B_6H_{12}$ (k=3) Isomer III

H	0.00000000	1.39274300	-3.24428100
H	0.00000000	3.05048000	-0.72810000
H	0.00000000	-3.05048000	-0.72810000
H	0.00000000	-1.39274300	-3.24428100
H	0.00000000	-1.33081900	3.14606300
H	0.00000000	1.33081900	3.14606300
B	0.00000000	0.90286700	-2.15981200
B	0.00000000	-0.90286700	-2.15981200
B	0.00000000	1.85821200	-0.72477300
B	0.00000000	-1.85821200	-0.72477300
B	0.00000000	0.84072200	0.70832700
B	0.00000000	-0.84072200	0.70832700
H	0.95963900	1.54752400	0.10585100
H	0.96071600	0.00000000	-2.14986200
H	0.95963900	-1.54752400	0.10585100
H	-0.95963900	1.54752400	0.10585100
H	-0.96071600	0.00000000	-2.14986200
H	-0.95963900	-1.54752400	0.10585100
C	0.00000000	0.68105900	2.27429400
C	0.00000000	-0.68105900	2.27429400

B_6H_{14} (k=4) Kekulé Structure K_2

0 1

H	0.00000000	1.40891800	-3.32224500
H	0.00000000	3.00525400	-0.78689600
H	0.00000000	-3.00525400	-0.78689600
H	0.00000000	-1.40891800	-3.32224500
H	0.00000000	-1.69684600	3.29075700
H	0.00000000	1.69684600	3.29075700
B	0.00000000	0.89935500	-2.24705500
B	0.00000000	-0.89935500	-2.24705500
B	0.00000000	1.81267600	-0.79308400
B	0.00000000	-1.81267600	-0.79308400
B	0.00000000	0.85658500	0.68542600
B	0.00000000	-0.85658500	0.68542600
B	0.00000000	0.90969000	2.39730200
B	0.00000000	-0.90969000	2.39730200
H	0.95715100	1.50212100	0.07107700
H	0.96140600	0.00000000	-2.25031100
H	0.95715100	-1.50212100	0.07107700
H	0.93205800	0.00000000	2.71359800
H	-0.95715100	1.50212100	0.07107700
H	-0.96140600	0.00000000	-2.25031100
H	-0.95715100	-1.50212100	0.07107700
H	-0.93205800	0.00000000	2.71359800

Benzocyclobutadiene Kekulé Structure K_3 : $C_{(8-2k)}B_{(2k)}H_{(6+2k)}$

$C_6B_2H_8$ (k=1) Isomer I

H	-1.54577300	-2.66444700	0.00000000
H	0.85506800	-2.67572600	0.00000000
H	-2.11706800	1.58181700	0.00000000
H	-2.95437500	-0.71753200	0.00000000
H	0.79539800	2.97617300	0.00000000
H	3.41442900	1.03716000	0.00000000
B	0.95535000	-0.32084300	0.00000000
B	2.23692000	0.89230800	0.00000000
H	1.99624700	0.01625200	0.93983400
H	1.99624700	0.01625200	-0.93983400
C	0.33967600	-1.72357700	0.00000000
C	-1.01758800	-1.71519200	0.00000000
C	-1.88846100	-0.52910500	0.00000000
C	-1.42930400	0.74323300	0.00000000
C	0.00000000	0.92149500	0.00000000
C	0.92875700	1.89860000	0.00000000

$C_6B_2H_8$ (k=1) Isomer II

H	-1.68297400	-2.94245500	0.00000000
H	1.38917200	-2.56292800	0.00000000
H	-2.00850200	1.59363900	0.00000000
H	-3.06542300	-0.57925300	0.00000000
H	1.14784400	2.98061100	0.00000000
H	3.11961500	0.92043300	0.00000000
B	-1.18896600	-1.86376000	0.00000000
B	0.63516100	-1.64831500	0.00000000
H	-0.27598500	-1.84682200	0.96098500
H	-0.27598500	-1.84682200	-0.96098500
C	-1.98100500	-0.55035400	0.00000000
C	-1.39573800	0.69822000	0.00000000
C	0.00000000	0.82667200	0.00000000
C	0.95338000	-0.16879000	0.00000000
C	2.04026300	0.93332500	0.00000000
C	1.11997800	1.90158900	0.00000000

$C_4B_4H_{10}$ (k=2) Isomer I

H	-1.88841500	-2.79212200	0.00000000
H	1.18054500	-2.71276400	0.00000000
H	-2.21107200	1.71266100	0.00000000
H	-3.14709700	-0.46533100	0.00000000
H	1.87239900	3.06283900	0.00000000
H	3.15939500	0.08447000	0.00000000
B	-1.27920800	-1.77155600	0.00000000
B	0.51721500	-1.72724200	0.00000000
B	0.00000000	0.97548100	0.00000000
B	1.47266500	1.94541000	0.00000000
H	-0.40084200	-1.90204700	0.96183300
H	-0.40084200	-1.90204700	-0.96183300
H	0.54951400	1.89342900	0.93575100
H	0.54951400	1.89342900	-0.93575100
C	-2.06142300	-0.38514900	0.00000000
C	-1.53791800	0.85991400	0.00000000
C	0.99257400	-0.25826200	0.00000000
C	2.13735700	0.45299900	0.00000000

$C_4B_4H_{10}$ (k=2) Isomer II

H	0.00000000	1.18065200	-2.89814100
H	0.00000000	2.63096600	-1.00542500
H	0.00000000	-2.63096600	-1.00542500
H	0.00000000	-1.18065200	-2.89814100
H	0.00000000	-1.72559100	3.14100900
H	0.00000000	1.72559100	3.14100900
B	0.00000000	0.84515800	0.54856000
B	0.00000000	-0.84515800	0.54856000
B	0.00000000	0.87174700	2.30991500
B	0.00000000	-0.87174700	2.30991500
H	0.93009600	1.26864100	1.43989700
H	0.93009600	-1.26864100	1.43989700
H	-0.93009600	1.26864100	1.43989700
H	-0.93009600	-1.26864100	1.43989700
C	0.00000000	1.56017500	-0.83077800
C	0.00000000	0.74232700	-1.90415700
C	0.00000000	-0.74232700	-1.90415700
C	0.00000000	-1.56017500	-0.83077800

$C_4B_4H_{10}$ (k=2) Isomer III

H	-1.76816700	-2.91528300	0.00000000
H	1.25031100	-2.88469100	0.00000000
H	-2.07383900	1.59231300	0.00000000
H	-3.04688500	-0.59111300	0.00000000
H	0.57875500	3.04746600	0.00000000
H	3.33704700	1.34964100	0.00000000
B	-1.17634100	-1.88409200	0.00000000
B	0.62174400	-1.87721300	0.00000000
B	1.10982800	-0.25398800	0.00000000
B	2.17226400	1.12741900	0.00000000
H	-0.24450600	-2.00523100	0.96254600
H	-0.24450600	-2.00523100	-0.96254600
H	1.99566900	0.11309200	0.92786600
H	1.99566900	0.11309200	-0.92786600
C	-1.96346500	-0.52391400	0.00000000
C	-1.42718000	0.71749400	0.00000000
C	0.00000000	0.92051600	0.00000000
C	0.82114000	1.99012500	0.00000000

$C_4B_4H_{10}$ (k=2) Isomer IV

H	0.00000000	1.53266300	-3.14313800
H	0.00000000	2.83740500	-0.44278900
H	0.00000000	-2.83740500	-0.44278900
H	0.00000000	-1.53266300	-3.14313800
H	0.00000000	-1.42767900	2.97095500
H	0.00000000	1.42767900	2.97095500
B	0.00000000	0.84645700	-2.17276900
B	0.00000000	-0.84645700	-2.17276900
B	0.00000000	1.65518900	-0.55439800
B	0.00000000	-1.65518900	-0.55439800
H	0.96247000	1.32063700	-1.41487300
H	0.96247000	-1.32063700	-1.41487300
H	-0.96247000	1.32063700	-1.41487300
H	-0.96247000	-1.32063700	-1.41487300
C	0.00000000	0.68144000	0.64145900
C	0.00000000	0.66621800	2.20530100
C	0.00000000	-0.66621800	2.20530100
C	0.00000000	-0.68144000	0.64145900

$C_2B_6H_{12}$ (k=3) Isomer I

H	3.19233100	-0.45311600	0.00000000
H	2.21909800	1.69015600	0.00000000
H	-0.93143400	-3.00584500	0.00000000
H	2.03547100	-2.79677600	0.00000000
H	-3.60444600	0.59880500	0.00000000
H	-1.46451100	3.29737500	0.00000000
B	1.37502700	-1.80728800	0.00000000
B	-0.41871700	-1.93299800	0.00000000
B	0.00000000	0.96750100	0.00000000
B	-1.07060000	-0.36486700	0.00000000
B	-1.35261900	2.11072400	0.00000000
B	-2.42276000	0.74962900	0.00000000
H	0.46986400	-1.97321600	0.96221100
H	0.46986400	-1.97321600	-0.96221100
H	-0.44501000	1.86283200	0.93039400
H	-1.99198500	-0.15147600	0.92463700
H	-0.44501000	1.86283200	-0.93039400
H	-1.99198500	-0.15147600	-0.92463700
C	2.10466800	-0.40059600	0.00000000
C	1.55134800	0.83053300	0.00000000

$C_2B_6H_{12}$ (k=3) Isomer II

H	3.33292500	-0.96794600	0.00000000
H	2.29969900	1.81944000	0.00000000
H	-1.48058100	-2.75151100	0.00000000
H	1.42152300	-3.24478100	0.00000000
H	-3.38354800	1.36424400	0.00000000
H	-0.66235100	3.12002900	0.00000000
B	2.15576100	-0.79443500	0.00000000
B	1.04782500	-2.11468400	0.00000000
B	1.54277100	0.90231000	0.00000000
B	-0.72919800	-1.83028300	0.00000000
B	-1.09264600	-0.17301000	0.00000000
B	-2.21365000	1.16658100	0.00000000
H	1.94239200	0.05347900	0.96254300
H	0.13892800	-2.04545200	0.96095000
H	1.94239200	0.05347900	-0.96254300
H	0.13892800	-2.04545200	-0.96095000
H	-2.00417800	0.18402800	0.92632600
H	-2.00417800	0.18402800	-0.92632600
C	0.00000000	1.02783000	0.00000000
C	-0.87271000	2.05450600	0.00000000

B_6H_{14} (k=4) Kekulé structure K_3

H	0.00000000	1.47425900	-3.27108400
H	0.00000000	3.01087300	-0.81413200
H	0.00000000	-3.01087300	-0.81413200
H	0.00000000	-1.47425900	-3.27108400
H	0.00000000	-1.72363900	3.26614000
H	0.00000000	1.72363900	3.26614000
B	0.00000000	0.86260200	-2.24984200
B	0.00000000	-0.86260200	-2.24984200
B	0.00000000	1.82424000	-0.72503000
B	0.00000000	-1.82424000	-0.72503000
B	0.00000000	0.86383300	0.68228100
B	0.00000000	-0.86383300	0.68228100
B	0.00000000	0.86244800	2.44256200
B	0.00000000	-0.86244800	2.44256200
H	0.96066100	1.38043100	-1.51276200
H	0.96066100	-1.38043100	-1.51276200
H	-0.96066100	1.38043100	-1.51276200
H	-0.96066100	-1.38043100	-1.51276200
H	0.92350000	1.27443200	1.54736900
H	0.92350000	-1.27443200	1.54736900
H	-0.92350000	1.27443200	1.54736900
H	-0.92350000	-1.27443200	1.54736900

Naphthalene Kekulé Structure K_1 : $C_{(10-2k)}B_{(2k)}H_{(8+2k)}$

$C_{10}H_8$ (k=0) - same energy for Kekulé structures K_1 and K_2

C	0.00000000	2.42389600	0.70584800
C	0.00000000	1.24066100	1.39693200
C	0.00000000	0.00000000	0.71398700
C	0.00000000	0.00000000	-0.71398700
C	0.00000000	1.24066100	-1.39693200
C	0.00000000	2.42389600	-0.70584800
H	0.00000000	-1.23924000	2.47981200
H	0.00000000	3.36455500	1.24026300
H	0.00000000	1.23924000	2.47981200
C	0.00000000	-1.24066100	1.39693200
C	0.00000000	-1.24066100	-1.39693200
H	0.00000000	1.23924000	-2.47981200
H	0.00000000	3.36455500	-1.24026300
C	0.00000000	-2.42389600	-0.70584800
C	0.00000000	-2.42389600	0.70584800
H	0.00000000	-1.23924000	-2.47981200
H	0.00000000	-3.36455500	-1.24026300
H	0.00000000	-3.36455500	1.24026300

$C_8B_2H_{10}$ (k=1) Isomer I

H	1.49004800	2.39485100	0.00000000
H	3.30821500	-1.47668400	0.00000000
H	1.08035200	-2.52341000	0.00000000
H	3.51507100	0.99519800	0.00000000
B	-1.14146100	1.66970900	0.00000000
H	-1.22671200	-2.42721200	0.00000000
H	-1.06085600	2.85467800	0.00000000
B	-2.68815700	0.78478500	0.00000000
H	-1.98060600	1.35551400	0.96419800
H	-1.98060600	1.35551400	-0.96419800
H	-3.36466100	-1.39906200	0.00000000
H	-3.74551300	1.32391200	0.00000000
C	0.00000000	-0.66386300	0.00000000
C	0.11099400	0.75267300	0.00000000
C	1.39581600	1.31625300	0.00000000
C	2.53686400	0.53283300	0.00000000
C	2.42045100	-0.85798900	0.00000000
C	1.17012300	-1.44413700	0.00000000
C	-2.49772500	-0.74780600	0.00000000
C	-1.28429700	-1.34225900	0.00000000

$C_8B_2H_{10}$ (k=1) Isomer II

B	0.00000000	0.90794800	0.00000000
B	0.00000000	-0.90794800	0.00000000
H	-1.58085300	-2.59166800	0.00000000
H	-3.50357000	1.18683200	0.00000000
H	-1.58085300	2.59166800	0.00000000
H	-3.50357000	-1.18683200	0.00000000
H	0.00000000	0.00000000	0.96098100
H	0.00000000	0.00000000	-0.96098100
H	1.58085300	2.59166800	0.00000000
H	1.58085300	-2.59166800	0.00000000
H	3.50357000	1.18683200	0.00000000
H	3.50357000	-1.18683200	0.00000000
C	-2.52153300	-0.72374800	0.00000000
C	-1.41936300	-1.51853700	0.00000000
C	1.41936300	1.51853700	0.00000000
C	2.52153300	0.72374800	0.00000000
C	1.41936300	-1.51853700	0.00000000
C	2.52153300	-0.72374800	0.00000000
C	-2.52153300	0.72374800	0.00000000
C	-1.41936300	1.51853700	0.00000000

$C_6B_4H_{12}$ (k=2) Isomer I

H	0.00000000	2.46254700	1.34749600
H	0.00000000	-1.24031600	3.48143200
H	0.00000000	-2.46254700	1.34749600
H	0.00000000	1.24031600	3.48143200
B	0.00000000	-1.60483700	-1.18028900
B	0.00000000	1.60483700	-1.18028900
H	0.00000000	-2.78904600	-1.06917600
H	0.00000000	2.78904600	-1.06917600
B	0.00000000	-0.84833200	-2.79535700
B	0.00000000	0.84833200	-2.79535700
H	-0.96400700	-1.30777900	-2.04309100
H	-0.96400700	1.30777900	-2.04309100
H	0.96400700	-1.30777900	-2.04309100
H	0.96400700	1.30777900	-2.04309100
H	0.00000000	-1.54215400	-3.76019500
H	0.00000000	1.54215400	-3.76019500
C	0.00000000	-0.71171700	0.10719000
C	0.00000000	0.71171700	0.10719000
C	0.00000000	1.38018400	1.33985400
C	0.00000000	0.69460000	2.54709900
C	0.00000000	-0.69460000	2.54709900
C	0.00000000	-1.38018400	1.33985400

$C_6B_4H_{12}$ (k=2) Isomer II

B	1.48973000	1.35489400	0.00000000
B	0.46942200	2.81795500	0.00000000
H	-2.56378900	1.04044000	0.00000000
H	0.91590100	3.91688000	0.00000000
H	2.67680800	1.39249100	0.00000000
H	-1.76968900	3.26760800	0.00000000
H	1.09003800	2.14808200	0.96492700
H	1.09003800	2.14808200	-0.96492700
B	-1.48973000	-1.35489400	0.00000000
H	2.56378900	-1.04044000	0.00000000
H	-2.67680800	-1.39249100	0.00000000
B	-0.46942200	-2.81795500	0.00000000
H	-1.09003800	-2.14808200	0.96492700
H	-1.09003800	-2.14808200	-0.96492700
H	1.76968900	-3.26760800	0.00000000
H	-0.91590100	-3.91688000	0.00000000
C	-1.02773400	2.47660200	0.00000000
C	-1.48973000	1.19903400	0.00000000
C	-0.69883300	-0.01434300	0.00000000
C	0.69883300	0.01434300	0.00000000
C	1.48973000	-1.19903400	0.00000000
C	1.02773400	-2.47660200	0.00000000

$C_6B_4H_{12}$ (k=2) Isomer III

B	0.00000000	1.32569000	-1.59795000
B	0.00000000	2.80942400	-0.60277800
H	0.00000000	1.07707900	2.46310900
H	0.00000000	3.90729200	-1.05310400
H	0.00000000	1.36032000	-2.78425500
H	0.00000000	3.28997300	1.63529200
H	0.96377000	2.14548700	-1.20826100
H	-0.96377000	2.14548700	-1.20826100
B	0.00000000	-1.32569000	-1.59795000
H	0.00000000	-1.36032000	-2.78425500
H	0.00000000	-1.07707900	2.46310900
B	0.00000000	-2.80942400	-0.60277800
H	0.96377000	-2.14548700	-1.20826100
H	-0.96377000	-2.14548700	-1.20826100
H	0.00000000	-3.90729200	-1.05310400
H	0.00000000	-3.28997300	1.63529200
C	0.00000000	0.00000000	-0.79575100
C	0.00000000	0.00000000	0.59805600
C	0.00000000	1.22394200	1.38696800
C	0.00000000	2.48798600	0.90506600
C	0.00000000	-1.22394200	1.38696800
C	0.00000000	-2.48798600	0.90506600

$C_6B_4H_{12}$ (k=2) Isomer IV

B	1.37677200	-1.79904500	0.00000000
B	-0.10819900	-0.98135400	0.00000000
B	0.00000000	0.84106900	0.00000000
B	2.80530500	-0.70986400	0.00000000
H	1.46002300	2.59693500	0.00000000
H	3.90469600	-1.16396800	0.00000000
H	1.54625900	-2.97636200	0.00000000
H	3.49900900	1.44471700	0.00000000
H	2.14244100	-1.33894800	0.96211000
H	-0.03279500	-0.08232100	0.95747900
H	2.14244100	-1.33894800	-0.96211000
H	-0.03279500	-0.08232100	-0.95747900
H	-1.80416100	-2.53249500	0.00000000
H	-1.45750000	2.61832000	0.00000000
H	-3.63844200	-1.01647000	0.00000000
H	-3.47919600	1.35806600	0.00000000
C	2.59213700	0.84344500	0.00000000
C	1.41682200	1.50868800	0.00000000
C	-1.57410600	-1.47189900	0.00000000
C	-2.63026800	-0.61356200	0.00000000
C	-1.37399700	1.53583200	0.00000000
C	-2.53381600	0.82412300	0.00000000

$C_4B_6H_{14}$ (k=3) Isomer I

B	1.08861000	-1.73475300	0.00000000
B	1.38120300	1.42342000	0.00000000
B	2.92667700	0.54007600	0.00000000
B	2.77599400	-1.14373100	0.00000000
H	1.37890300	2.61273600	0.00000000
H	3.66747000	-1.92916700	0.00000000
H	0.88823700	-2.90595000	0.00000000
H	3.93752900	1.16373700	0.00000000
H	1.97861400	-1.51612500	0.96330600
H	2.19785800	1.05289700	0.96443100
H	1.97861400	-1.51612500	-0.96330600
H	2.19785800	1.05289700	-0.96443100
B	-1.52533000	-1.41729500	0.00000000
H	-1.67242300	-2.59488300	0.00000000
H	-0.91676100	2.59340800	0.00000000
B	-2.91666600	-0.29562000	0.00000000
H	-2.29699600	-0.95169400	0.96416600
H	-2.29699600	-0.95169400	-0.96416600
H	-4.04791000	-0.65355000	0.00000000
H	-3.19190800	1.97453200	0.00000000
C	-0.12721300	-0.73645900	0.00000000
C	0.00000000	0.64807400	0.00000000
C	-1.15333300	1.53432200	0.00000000
C	-2.46187600	1.17214600	0.00000000

$C_4B_6H_{14}$ (k=3) Isomer II

B	0.00000000	1.54851300	-1.75496300
B	0.00000000	0.00000000	-1.05654700
B	0.00000000	0.00000000	0.76651100
B	0.00000000	2.91190800	-0.59000800
H	0.00000000	1.33556700	2.60904500
H	0.00000000	4.03881100	-0.97036600
H	0.00000000	1.79471300	-2.91834600
H	0.00000000	3.45327900	1.61168200
H	0.96214100	2.28935900	-1.24997300
H	0.95676700	0.00000000	-0.17258000
H	-0.96214100	2.28935900	-1.24997300
H	-0.95676700	0.00000000	-0.17258000
B	0.00000000	-1.54851300	-1.75496300
H	0.00000000	-1.79471300	-2.91834600
H	0.00000000	-1.33556700	2.60904500
B	0.00000000	-2.91190800	-0.59000800
H	0.96214100	-2.28935900	-1.24997300
H	-0.96214100	-2.28935900	-1.24997300
H	0.00000000	-4.03881100	-0.97036600
H	0.00000000	-3.45327900	1.61168200
C	0.00000000	2.59360200	0.94476700
C	0.00000000	1.37193200	1.52030800
C	0.00000000	-1.37193200	1.52030800
C	0.00000000	-2.59360200	0.94476700

$C_4B_6H_{14}$ (k=3) Isomer III

B	-1.46182900	1.70556400	0.00000000
B	0.03834100	0.91255000	0.00000000
B	-0.03834100	-0.91255000	0.00000000
B	-2.88450900	0.61331700	0.00000000
H	-1.49135000	-2.66341400	0.00000000
H	-3.98523600	1.06277200	0.00000000
H	-1.63753200	2.88215600	0.00000000
H	-3.54602500	-1.55151500	0.00000000
H	-2.21678000	1.23935000	0.96288200
H	0.00000000	0.00000000	0.95474700
H	-2.21678000	1.23935000	-0.96288200
H	0.00000000	0.00000000	-0.95474700
B	1.46182900	-1.70556400	0.00000000
H	1.49135000	2.66341400	0.00000000
H	1.63753200	-2.88215600	0.00000000
B	2.88450900	-0.61331700	0.00000000
H	2.21678000	-1.23935000	0.96288200
H	2.21678000	-1.23935000	-0.96288200
H	3.54602500	1.55151500	0.00000000
H	3.98523600	-1.06277200	0.00000000
C	-2.65098200	-0.93280500	0.00000000
C	-1.46182900	-1.57532500	0.00000000
C	1.46182900	1.57532500	0.00000000
C	2.65098200	0.93280500	0.00000000

$C_4B_6H_{14}$ (k=3) Isomer IV

B	0.00000000	0.92971600	0.11218700
B	0.00000000	-0.92971600	0.11218700
H	0.00000000	-2.59310900	1.70524000
H	0.00000000	1.19753500	3.65645300
H	0.00000000	2.59310900	1.70524000
H	0.00000000	-1.19753500	3.65645300
H	0.96356600	0.00000000	0.06030700
H	-0.96356600	0.00000000	0.06030700
B	0.00000000	1.79303600	-1.36831600
B	0.00000000	-1.79303600	-1.36831600
H	0.00000000	2.99720200	-1.43773000
H	0.00000000	-2.99720200	-1.43773000
B	0.00000000	0.86208300	-2.93286200
B	0.00000000	-0.86208300	-2.93286200
H	0.97301700	1.37700600	-2.18721300
H	0.97301700	-1.37700600	-2.18721300
H	-0.97301700	1.37700600	-2.18721300
H	-0.97301700	-1.37700600	-2.18721300
H	0.00000000	1.50156300	-3.95498100
H	0.00000000	-1.50156300	-3.95498100
C	0.00000000	1.50876600	1.54614400
C	0.00000000	0.72115400	2.66887200
C	0.00000000	-0.72115400	2.66887200
C	0.00000000	-1.50876600	1.54614400

$C_2B_8H_{16}$ (k=4) Isomer I

B	-1.31383300	1.57914000	0.00000000
B	-1.31383300	-1.57914000	0.00000000
B	-2.94094300	-0.84403200	0.00000000
B	-2.94094300	0.84403200	0.00000000
H	-1.22430700	-2.76381600	0.00000000
H	-3.89109600	1.55706200	0.00000000
H	-1.22430700	2.76381600	0.00000000
H	-3.89109600	-1.55706200	0.00000000
H	-2.16862100	1.28048100	0.96337800
H	-2.16862100	-1.28048100	0.96337800
H	-2.16862100	1.28048100	-0.96337800
H	-2.16862100	-1.28048100	-0.96337800
B	1.31383300	1.57914000	0.00000000
B	1.31383300	-1.57914000	0.00000000
H	1.22430700	2.76381600	0.00000000
H	1.22430700	-2.76381600	0.00000000
B	2.94094300	0.84403200	0.00000000
B	2.94094300	-0.84403200	0.00000000
H	2.16862100	1.28048100	0.96337800
H	2.16862100	-1.28048100	0.96337800
H	2.16862100	1.28048100	-0.96337800
H	2.16862100	-1.28048100	-0.96337800
H	3.89109600	1.55706200	0.00000000
H	3.89109600	-1.55706200	0.00000000
C	0.00000000	0.69064800	0.00000000
C	0.00000000	-0.69064800	0.00000000

$C_2B_8H_{16}$ (k=4) Isomer II

B	1.30210600	-1.91797500	0.00000000
B	-0.13466900	-0.98084000	0.00000000
B	0.00000000	0.84542700	0.00000000
B	1.54410600	1.59292800	0.00000000
B	3.02813000	0.58741800	0.00000000
B	2.91159200	-1.12201100	0.00000000
H	1.67846400	2.77675400	0.00000000
H	3.87398300	-1.82248100	0.00000000
H	1.28854100	-3.10852000	0.00000000
H	4.07352900	1.15600600	0.00000000
H	2.13733100	-1.56699000	0.96060500
H	2.31510200	1.13481000	0.96139600
H	-0.03897100	-0.08578700	0.95230300
H	2.13733100	-1.56699000	-0.96060500
H	2.31510200	1.13481000	-0.96139600
H	-0.03897100	-0.08578700	-0.95230300
B	-1.72993400	-1.56763600	0.00000000
H	-2.05077500	-2.71294600	0.00000000
H	-1.22682300	2.75646900	0.00000000
B	-3.01594300	-0.32019600	0.00000000
H	-2.42952200	-1.01546300	0.96267900
H	-2.42952200	-1.01546300	-0.96267900
H	-4.16418800	-0.62952200	0.00000000
H	-3.40367500	1.91315900	0.00000000
C	-1.33320900	1.67286100	0.00000000
C	-2.59410500	1.18586700	0.00000000

$B_{10}H_{18}$ (k=5) - Kekulé Structure K_1

B	-1.50496200	1.74986000	0.00000000
B	0.00000000	0.91728300	0.00000000
B	0.00000000	-0.91728300	0.00000000
B	-1.50496200	-1.74986000	0.00000000
B	-3.06005500	-0.85558400	0.00000000
B	-3.06005500	0.85558400	0.00000000
H	-1.56750400	-2.93907600	0.00000000
H	-4.06214000	1.49753800	0.00000000
H	-1.56750400	2.93907600	0.00000000
H	-4.06214000	-1.49753800	0.00000000
H	-2.30830000	1.34504700	0.96095200
H	-2.30830000	-1.34504700	0.96095200
H	0.00000000	0.00000000	0.94969300
H	-2.30830000	1.34504700	-0.96095200
H	-2.30830000	-1.34504700	-0.96095200
H	0.00000000	0.00000000	-0.94969300
B	1.50496200	1.74986000	0.00000000
B	1.50496200	-1.74986000	0.00000000
H	1.56750400	2.93907600	0.00000000
H	1.56750400	-2.93907600	0.00000000
B	3.06005500	0.85558400	0.00000000
B	3.06005500	-0.85558400	0.00000000
H	2.30830000	1.34504700	0.96095200
H	2.30830000	-1.34504700	0.96095200
H	2.30830000	1.34504700	-0.96095200
H	2.30830000	-1.34504700	-0.96095200
H	4.06214000	1.49753800	0.00000000
H	4.06214000	-1.49753800	0.00000000

Naphthalene Kekulé Structure K_2 : $C_{(10-2k)}B_{(2k)}H_{(8+2k)}$

$C_8B_2H_{10}$ (k=1) Isomer I – same structure and energy as Isomer I in K_1 (see above)

$C_8B_2H_{10}$ (k=1) Isomer II – same structure and energy as Isomer II in K_1 (see above)

$C_8B_2H_{10}$ (k=1) Isomer III

B	0.00000000	0.90248800	2.56883400
B	0.00000000	-0.90248800	2.56883400
H	0.00000000	2.55899500	1.02215600
H	0.00000000	-1.55065600	3.56256800
H	0.00000000	-2.55899500	1.02215600
H	0.00000000	1.55065600	3.56256800
H	-0.96347100	0.00000000	2.67912500
H	0.96347100	0.00000000	2.67912500
H	0.00000000	-2.48291200	-1.28134400
H	0.00000000	2.48291200	-1.28134400
H	0.00000000	-1.24532100	-3.39212800
H	0.00000000	1.24532100	-3.39212800
C	0.00000000	-1.48198200	1.15863700
C	0.00000000	-0.73472900	-0.00096400
C	0.00000000	-1.40050000	-1.28138200
C	0.00000000	-0.71564700	-2.44871500
C	0.00000000	0.71564700	-2.44871500
C	0.00000000	1.40050000	-1.28138200
C	0.00000000	0.73472900	-0.00096400
C	0.00000000	1.48198200	1.15863700

$C_6B_4H_{12}$ (k=2) Isomer I

H	0.00000000	2.46275800	1.34701600
H	0.00000000	-1.24006100	3.48173600
H	0.00000000	-2.46275800	1.34701600
H	0.00000000	1.24006100	3.48173600
B	0.00000000	-1.60447300	-1.18051500
B	0.00000000	1.60447300	-1.18051500
H	0.00000000	-2.78867400	-1.06896500
H	0.00000000	2.78867400	-1.06896500
B	0.00000000	-0.84853600	-2.79571400
B	0.00000000	0.84853600	-2.79571400
H	-0.96405300	-1.30631300	-2.04264900
H	-0.96405300	1.30631300	-2.04264900
H	0.96405300	-1.30631300	-2.04264900
H	0.96405300	1.30631300	-2.04264900
H	0.00000000	-1.54364800	-3.75959600
H	0.00000000	1.54364800	-3.75959600
C	0.00000000	-0.71175600	0.10752700
C	0.00000000	-1.38013600	1.33978400
C	0.00000000	-0.69450700	2.54706600
C	0.00000000	0.69450700	2.54706600
C	0.00000000	1.38013600	1.33978400
C	0.00000000	0.71175600	0.10752700

$C_6B_4H_{12}$ (k=2) Isomer II

B	0.00000000	0.76700900	0.00000000
B	1.67420600	1.46367700	0.00000000
H	1.89244900	2.63137700	0.00000000
H	3.29438100	-1.67367200	0.00000000
H	1.09369900	-2.58789600	0.00000000
H	3.81325600	0.65405800	0.00000000
H	0.82584700	1.22634200	0.96340000
H	0.82584700	1.22634200	-0.96340000
B	-1.37751100	-1.53546300	0.00000000
H	-1.46645600	-2.72157500	0.00000000
H	-1.23911400	2.70175900	0.00000000
B	-2.81791900	-0.47703100	0.00000000
H	-2.17238000	-1.10752500	0.96419700
H	-2.17238000	-1.10752500	-0.96419700
H	-3.91699800	-0.92744500	0.00000000
H	-3.39803700	1.72855900	0.00000000
C	-2.53915700	1.06153700	0.00000000
C	-1.31027100	1.61550400	0.00000000
C	-0.01203000	-0.78312800	0.00000000
C	1.15389000	-1.50237300	0.00000000
C	2.48035100	-0.95550400	0.00000000
C	2.76488500	0.37500400	0.00000000

$C_6B_4H_{12}$ (k=2) Isomer III

B	2.55897500	1.04723100	0.00000000
B	2.68998200	-0.73811500	0.00000000
H	0.89925700	2.58531400	0.00000000
H	3.71986200	-1.32750000	0.00000000
H	1.26250000	-2.49700100	0.00000000
H	3.49422500	1.77744900	0.00000000
H	2.75731500	0.15919700	0.96373600
H	2.75731500	0.15919700	-0.96373600
B	-1.19068400	-1.66371900	0.00000000
H	-1.10882300	-2.84799500	0.00000000
H	-1.26580600	2.43652700	0.00000000
B	-2.72259600	-0.78193200	0.00000000
H	-2.02868100	-1.36648600	0.96474500
H	-2.02868100	-1.36648600	-0.96474500
H	-3.78665100	-1.30712100	0.00000000
H	-3.38861200	1.40694800	0.00000000
C	-2.52120300	0.75596800	0.00000000
C	-1.31461300	1.35178800	0.00000000
C	0.00000000	0.69871800	0.00000000
C	1.10285700	1.51898300	0.00000000
C	0.09801900	-0.76776200	0.00000000
C	1.30800500	-1.41259000	0.00000000

$C_6B_4H_{12}$ (k=2) Isomer IV

B	1.66509800	-1.56612900	0.00000000
B	-0.00680500	-0.88228500	0.00000000
H	1.11486200	2.48058700	0.00000000
H	3.81160900	-0.78399200	0.00000000
H	1.86822200	-2.73563200	0.00000000
H	3.30759600	1.55021600	0.00000000
H	0.78251700	-1.33395600	0.96044700
H	0.78251700	-1.33395600	-0.96044700
B	-1.46566200	-1.73396400	0.00000000
H	-1.61211700	-2.91403100	0.00000000
H	-1.14157900	2.51162800	0.00000000
B	-2.85015200	-0.61130600	0.00000000
H	-2.22937700	-1.29436700	0.96223400
H	-2.22937700	-1.29436700	-0.96223400
H	-3.97984900	-0.98200800	0.00000000
H	-3.31279200	1.61505600	0.00000000
C	-2.49343400	0.90325900	0.00000000
C	-1.24772800	1.42825900	0.00000000
C	0.00000000	0.67986200	0.00000000
C	1.17464200	1.39496700	0.00000000
C	2.48841100	0.83793800	0.00000000
C	2.76567100	-0.49707900	0.00000000

$C_6B_4H_{12}$ (k=2) Isomer V

B	0.00000000	1.62639500	-1.49998100
B	0.00000000	0.84483600	0.11349700
B	0.00000000	-0.84483600	0.11349700
B	0.00000000	-1.62639500	-1.49998100
H	0.00000000	-2.80999900	-1.62934200
H	0.00000000	1.16349700	-3.71405800
H	0.00000000	2.80999900	-1.62934200
H	0.00000000	-1.16349700	-3.71405800
H	-0.96298100	-1.34350100	-0.64874200
H	-0.96298100	1.34350100	-0.64874200
H	0.96298100	-1.34350100	-0.64874200
H	0.96298100	1.34350100	-0.64874200
H	0.00000000	2.59479100	1.66422600
H	0.00000000	-2.59479100	1.66422600
H	0.00000000	1.19271600	3.59597000
H	0.00000000	-1.19271600	3.59597000
C	0.00000000	1.51944800	1.51455500
C	0.00000000	0.73360800	2.61180000
C	0.00000000	-0.73360800	2.61180000
C	0.00000000	-1.51944800	1.51455500
C	0.00000000	0.67674800	-2.74083700
C	0.00000000	-0.67674800	-2.74083700

$C_4B_6H_{14}$ (k=3) Isomer I

B	0.00000000	0.89167000	2.69335600
B	0.00000000	-0.89167000	2.69335600
H	0.00000000	2.54333400	1.14865300
H	0.00000000	-1.55840300	3.67509200
H	0.00000000	-2.54333400	1.14865300
H	0.00000000	1.55840300	3.67509200
H	-0.96379600	0.00000000	2.83319500
H	0.96379600	0.00000000	2.83319500
B	0.00000000	-1.60508600	-1.21949600
B	0.00000000	1.60508600	-1.21949600
H	0.00000000	-2.78943500	-1.12090300
H	0.00000000	2.78943500	-1.12090300
B	0.00000000	-0.84948700	-2.83005800
B	0.00000000	0.84948700	-2.83005800
H	-0.96437500	-1.30551900	-2.07704200
H	-0.96437500	1.30551900	-2.07704200
H	0.96437500	-1.30551900	-2.07704200
H	0.96437500	1.30551900	-2.07704200
H	0.00000000	-1.54387000	-3.79411700
H	0.00000000	1.54387000	-3.79411700
C	0.00000000	-0.73752200	0.10087100
C	0.00000000	-1.46440100	1.26465500
C	0.00000000	0.73752200	0.10087100
C	0.00000000	1.46440100	1.26465500

$C_4B_6H_{14}$ (k=3) Isomer II

B	1.82814100	-1.38039700	0.00000000
B	0.10709600	-0.81196200	0.00000000
H	0.99890200	2.61153500	0.00000000
H	3.91908500	-0.44867700	0.00000000
H	2.11605800	-2.53225600	0.00000000
H	3.24834600	1.84409200	0.00000000
H	0.93904500	-1.20679800	0.95893100
H	0.93904500	-1.20679800	-0.95893100
B	-1.22317600	-1.87829400	0.00000000
B	-1.37596800	1.49406600	0.00000000
H	-1.11814000	-3.06485600	0.00000000
H	-1.42069800	2.68384500	0.00000000
B	-2.84794500	-1.13425600	0.00000000
B	-2.91451500	0.57186300	0.00000000
H	-2.07427200	-1.59279200	0.96086600
H	-2.19416100	1.07151800	0.96403500
H	-2.07427200	-1.59279200	-0.96086600
H	-2.19416100	1.07151800	-0.96403500
H	-3.81240100	-1.83151700	0.00000000
H	-3.92382500	1.20047500	0.00000000
C	0.00000000	0.74582500	0.00000000
C	1.12391400	1.53161400	0.00000000
C	2.48448900	1.07253000	0.00000000
C	2.85547900	-0.23523600	0.00000000

$C_4B_6H_{14}$ (k=3) Isomer III

B	1.54087600	-1.73470900	0.00000000
B	-0.03952600	-0.86570000	0.00000000
B	2.62650700	0.97182000	0.00000000
B	2.94021900	-0.78802000	0.00000000
H	1.01468100	2.54430800	0.00000000
H	4.06059900	-1.18394700	0.00000000
H	1.50459100	-2.92284600	0.00000000
H	3.51786300	1.75857600	0.00000000
H	2.88614200	0.09816900	0.96302100
H	0.72788500	-1.36545200	0.95990700
H	2.88614200	0.09816900	-0.96302100
H	0.72788500	-1.36545200	-0.95990700
B	-1.52951000	-1.68529600	0.00000000
H	-1.67725600	-2.86509700	0.00000000
H	-1.15179600	2.54938300	0.00000000
B	-2.89564300	-0.55372400	0.00000000
H	-2.28939600	-1.25225500	0.96254200
H	-2.28939600	-1.25225500	-0.96254200
H	-4.03165400	-0.90370900	0.00000000
H	-3.32305400	1.67858200	0.00000000
C	-2.51282400	0.95630400	0.00000000
C	-1.26491800	1.46719700	0.00000000
C	0.00000000	0.72414900	0.00000000
C	1.14810000	1.46267900	0.00000000

$C_4B_6H_{14}$ (k=3) Isomer IV

B	1.39799100	-1.80072300	0.00000000
B	-0.15421200	-0.91024600	0.00000000
B	0.00000000	0.78026000	0.00000000
B	1.68673800	1.41141000	0.00000000
H	1.91057500	2.58099900	0.00000000
H	3.64991200	-1.55892900	0.00000000
H	1.42268400	-2.99067300	0.00000000
H	3.86150600	0.76651800	0.00000000
H	0.82500400	1.18722600	0.96193600
H	0.54071300	-1.45773000	0.96045900
H	0.82500400	1.18722600	-0.96193600
H	0.54071300	-1.45773000	-0.96045900
B	-1.72702600	-1.55613300	0.00000000
H	-2.05851100	-2.69902200	0.00000000
H	-1.13382200	2.79133600	0.00000000
B	-2.97258400	-0.27211200	0.00000000
H	-2.41517900	-1.00058000	0.96264400
H	-2.41517900	-1.00058000	-0.96264400
H	-4.12919900	-0.55018900	0.00000000
H	-3.32920400	1.96805800	0.00000000
C	-2.52411500	1.23570200	0.00000000
C	-1.26218600	1.70937100	0.00000000
C	2.72776900	-0.98122500	0.00000000
C	2.85027300	0.36478400	0.00000000

$C_4B_6H_{14}$ (k=3) Isomer V

B	0.00000000	1.76960100	-1.42031100
B	0.00000000	0.85617100	0.13087200
B	0.00000000	-0.85617100	0.13087200
B	0.00000000	-1.76960100	-1.42031100
B	0.00000000	-0.89878300	-2.89117500
B	0.00000000	0.89878300	-2.89117500
H	0.00000000	-2.96038100	-1.36730800
H	0.00000000	1.43612900	-3.95297800
H	0.00000000	2.96038100	-1.36730800
H	0.00000000	-1.43612900	-3.95297800
H	-0.96111600	0.00000000	-2.93305000
H	-0.96162000	-1.37610100	-0.63168900
H	-0.96162000	1.37610100	-0.63168900
H	0.96111600	0.00000000	-2.93305000
H	0.96162000	-1.37610100	-0.63168900
H	0.96162000	1.37610100	-0.63168900
H	0.00000000	2.59175100	1.68801800
H	0.00000000	-2.59175100	1.68801800
H	0.00000000	1.19485100	3.62463400
H	0.00000000	-1.19485100	3.62463400
C	0.00000000	1.51579300	1.54342300
C	0.00000000	0.73515800	2.64109900
C	0.00000000	-0.73515800	2.64109900
C	0.00000000	-1.51579300	1.54342300

$C_2B_8H_{16}$ (k=4) Isomer I

B	-0.07326800	-0.80584200	0.00000000
B	-1.71810600	-1.56638100	0.00000000
B	-3.05102300	-0.52628600	0.00000000
B	-2.61842600	1.20390200	0.00000000
H	-1.76779200	-2.75411400	0.00000000
H	-3.45048100	2.05348500	0.00000000
H	-0.90344400	2.66154500	0.00000000
H	-4.19549700	-0.84616800	0.00000000
H	-2.93938600	0.35886700	0.96292700
H	-0.88520600	-1.25093800	0.95814700
H	-2.93938600	0.35886700	-0.96292700
H	-0.88520600	-1.25093800	-0.95814700
B	1.38545600	1.53467100	0.00000000
B	1.30267100	-1.82896900	0.00000000
H	1.41650200	2.72419400	0.00000000
H	1.21614200	-3.01669800	0.00000000
B	2.94062500	0.64633700	0.00000000
B	2.91202500	-1.06060000	0.00000000
H	2.20672900	1.12578100	0.96423800
H	2.14505600	-1.53332300	0.96112800
H	2.20672900	1.12578100	-0.96423800
H	2.14505600	-1.53332300	-0.96112800
H	3.93479800	1.29816200	0.00000000
H	3.88897300	-1.73959200	0.00000000
C	0.00000000	0.78270300	0.00000000
C	-1.09889300	1.58967300	0.00000000

$C_2B_8H_{16}$ (k=4) Isomer II

B	0.00000000	1.60448100	1.61992900
B	0.00000000	0.85403000	-0.01387300
B	0.00000000	-0.85403000	-0.01387300
B	0.00000000	-1.60448100	1.61992900
H	0.00000000	-2.78821300	1.74382600
H	0.00000000	1.16973400	3.84585800
H	0.00000000	2.78821300	1.74382600
H	0.00000000	-1.16973400	3.84585800
H	0.95906100	-1.32425500	0.74507500
H	0.95906100	1.32425500	0.74507500
H	-0.95906100	-1.32425500	0.74507500
H	-0.95906100	1.32425500	0.74507500
B	0.00000000	1.76864100	-1.45812500
B	0.00000000	-1.76864100	-1.45812500
H	0.00000000	2.95842700	-1.51854200
H	0.00000000	-2.95842700	-1.51854200
B	0.00000000	0.85849300	-3.00549200
B	0.00000000	-0.85849300	-3.00549200
H	0.96107400	1.35526000	-2.25920100
H	0.96107400	-1.35526000	-2.25920100
H	-0.96107400	1.35526000	-2.25920100
H	-0.96107400	-1.35526000	-2.25920100
H	0.00000000	1.49076200	-4.01399600
H	0.00000000	-1.49076200	-4.01399600
C	0.00000000	0.67543500	2.87648500
C	0.00000000	-0.67543500	2.87648500

$C_2B_8H_{16}$ (k=4) Isomer III

B	-1.65614900	1.52667800	0.00000000
B	0.00000000	0.79694000	0.00000000
B	0.19333000	-0.91119600	0.00000000
B	-1.27017300	-1.95735800	0.00000000
B	-2.83680500	-1.26719700	0.00000000
B	-3.03454400	0.51565700	0.00000000
H	-1.09730800	-3.13584700	0.00000000
H	-4.14514500	0.94304300	0.00000000
H	-1.72479100	2.71667300	0.00000000
H	-3.82531200	-1.93017500	0.00000000
H	-2.98896500	-0.38531700	0.96110600
H	-0.49404000	-1.49007900	0.95855700
H	-0.83982300	1.20338000	0.96001200
H	-2.98896500	-0.38531700	-0.96110600
H	-0.49404000	-1.49007900	-0.95855700
H	-0.83982300	1.20338000	-0.96001200
B	1.78947400	-1.51468000	0.00000000
H	1.11366300	2.81009400	0.00000000
H	2.13301400	-2.65395400	0.00000000
B	3.01275200	-0.21602300	0.00000000
H	2.47028100	-0.95522600	0.96298500
H	2.47028100	-0.95522600	-0.96298500
H	3.32196600	2.03141000	0.00000000
H	4.17461700	-0.47025600	0.00000000
C	2.53226900	1.28254600	0.00000000
C	1.26189200	1.73068600	0.00000000

$B_{10}H_{18}$ (k=5) – Kekulé structure K_2

B	0.00000000	1.74417700	1.54761600
B	0.00000000	0.86509000	-0.03241200
B	0.00000000	-0.86509000	-0.03241200
B	0.00000000	-1.74417700	1.54761600
B	0.00000000	-0.89571900	3.03332000
B	0.00000000	0.89571900	3.03332000
H	0.00000000	-2.93407300	1.49697700
H	0.00000000	1.44856000	4.08729300
H	0.00000000	2.93407300	1.49697700
H	0.00000000	-1.44856000	4.08729300
H	0.96123500	0.00000000	3.08978900
H	0.95681700	-1.34991800	0.73359200
H	0.95681700	1.34991800	0.73359200
H	-0.96123500	0.00000000	3.08978900
H	-0.95681700	-1.34991800	0.73359200
H	-0.95681700	1.34991800	0.73359200
B	0.00000000	1.76159100	-1.49488700
B	0.00000000	-1.76159100	-1.49488700
H	0.00000000	2.95131300	-1.55332800
H	0.00000000	-2.95131300	-1.55332800
B	0.00000000	0.85796800	-3.04383800
B	0.00000000	-0.85796800	-3.04383800
H	0.96130600	1.35008000	-2.29347500
H	0.96130600	-1.35008000	-2.29347500
H	-0.96130600	1.35008000	-2.29347500
H	-0.96130600	-1.35008000	-2.29347500
H	0.00000000	1.49355800	-4.04995300
H	0.00000000	-1.49355800	-4.04995300

Azulene $C_{(10-2k)}B_{(2k)}H_{(8+2k)}$

$C_{10}H_8$ (k=0)

C	0.00000000	0.00000000	-2.49343800
C	0.00000000	1.26113200	-1.90366000
C	0.00000000	1.58900700	-0.55026500
C	0.00000000	-1.26113200	-1.90366000
C	0.00000000	0.74773500	0.55154300
C	0.00000000	-1.58900700	-0.55026500
C	0.00000000	-0.74773500	0.55154300
H	0.00000000	0.00000000	-3.57782100
H	0.00000000	2.09910300	-2.58997200
H	0.00000000	2.65050500	-0.32290000
H	0.00000000	-2.09910300	-2.58997200
H	0.00000000	-2.65050500	-0.32290000
C	0.00000000	1.14577400	1.89503400
H	0.00000000	2.16779100	2.24004300
C	0.00000000	0.00000000	2.69888800
H	0.00000000	0.00000000	3.77895200
C	0.00000000	-1.14577400	1.89503400
H	0.00000000	-2.16779100	2.24004300

$C_8B_2H_{10}$ (k=1) Isomer I

H	-2.99402400	2.08304200	0.00000000
H	-3.33631300	-0.17340500	0.00000000
H	-1.82808200	-1.93161200	0.00000000
H	-1.05491900	3.32366200	0.00000000
H	1.15022400	2.62225900	0.00000000
H	0.34730700	-3.07735700	0.00000000
H	2.84488800	-2.70912100	0.00000000
H	3.62616000	0.10938500	0.00000000
B	0.89436600	0.38168400	0.00000000
B	2.54159000	-0.37236100	0.00000000
H	1.87423800	0.22782300	0.95154400
H	1.87423800	0.22782300	-0.95154400
C	0.40923000	1.82695700	0.00000000
C	-0.88774800	2.25047300	0.00000000
C	-2.08884800	1.48575200	0.00000000
C	-2.29518300	0.13001100	0.00000000
C	-1.37116500	-0.94449200	0.00000000
C	0.00000000	-0.89890600	0.00000000
C	0.80627100	-2.09337700	0.00000000
C	2.14686200	-1.88127100	0.00000000

$C_8B_2H_{10}$ (k=1) Isomer II

H	-2.88976800	-2.21037600	0.00000000
H	-0.82515100	-3.27297800	0.00000000
H	1.30574500	-2.31511500	0.00000000
H	-3.35501600	0.08472700	0.00000000
H	-1.89694900	1.86780000	0.00000000
H	3.30594300	-0.60885400	0.00000000
H	3.07248700	2.74454800	0.00000000
H	0.17831700	3.03677700	0.00000000
B	2.22454500	1.91657600	0.00000000
B	2.37676200	0.12827000	0.00000000
H	2.57156300	1.00303700	0.95099600
H	2.57156300	1.00303700	-0.95099600
C	0.70110000	2.08887700	0.00000000
C	0.00000000	0.90821900	0.00000000
C	0.86750900	-0.28688100	0.00000000
C	0.49205800	-1.59720900	0.00000000
C	-0.80970400	-2.18947200	0.00000000
C	-2.01767100	-1.56652100	0.00000000
C	-2.30081400	-0.16652100	0.00000000
C	-1.44002200	0.88337000	0.00000000

$C_8B_2H_{10}$ (k=1) Isomer III

H	-3.06463300	2.13633700	0.00000000
H	-3.34118600	-0.12582400	0.00000000
H	-1.83417200	-1.83584100	0.00000000
H	-0.90675200	3.61158500	0.00000000
H	1.71437600	2.45782800	0.00000000
H	0.26815700	-3.12752900	0.00000000
H	2.86531300	-2.59370600	0.00000000
H	3.18707100	0.06907600	0.00000000
B	-0.83548400	2.42600900	0.00000000
B	0.79862600	1.70202200	0.00000000
H	-0.03512700	2.09167700	0.97115400
H	-0.03512700	2.09167700	-0.97115400
C	-2.13785300	1.56931900	0.00000000
C	-2.31397800	0.22701100	0.00000000
C	-1.36072500	-0.85828000	0.00000000
C	0.00000000	-0.90074600	0.00000000
C	0.73098900	-2.15282700	0.00000000
C	2.06113900	-1.87340000	0.00000000
C	2.23221100	-0.43552100	0.00000000
C	1.01594400	0.18853700	0.00000000

$C_8B_2H_{10}$ (k=1) Isomer IV

C	0.68822000	-1.60188100	0.00000000
C	-2.38754200	0.01237700	0.00000000
C	0.94006000	-0.27311500	0.00000000
C	-1.43059100	0.97626100	0.00000000
C	0.00000000	0.90124800	0.00000000
H	-3.20132600	-2.22445400	0.00000000
H	-0.69157500	-3.54853200	0.00000000
H	1.58031700	-2.22410000	0.00000000
H	-3.40521400	0.39042700	0.00000000
H	-1.78065600	2.00610700	0.00000000
C	2.27679100	0.29167900	0.00000000
H	3.17788800	-0.30119100	0.00000000
C	2.18314300	1.64710300	0.00000000
H	3.00776100	2.34376100	0.00000000
C	0.79148700	2.02044200	0.00000000
H	0.42290200	3.03549000	0.00000000
B	-2.24660000	-1.51840200	0.00000000
B	-0.67188100	-2.36124700	0.00000000
H	-1.44354900	-1.96197100	0.97106000
H	-1.44354900	-1.96197100	-0.97106000

$C_8B_2H_{10}$ (k=1) Isomer V

H	-3.03937500	-2.05358400	0.00000000
H	-1.19681800	-3.36886100	0.00000000
H	1.34856000	-2.79631100	0.00000000
H	-3.33552900	0.18895300	0.00000000
H	-1.88573200	1.94402700	0.00000000
H	3.43129200	-0.00293800	0.00000000
H	2.96404300	2.52886600	0.00000000
H	0.40511300	3.10172800	0.00000000
B	0.51653500	-1.94776200	0.00000000
B	1.03827700	-0.25510500	0.00000000
H	0.85779300	-1.09827400	0.97434600
H	0.85779300	-1.09827400	-0.97434600
C	-0.98109800	-2.30430900	0.00000000
C	-2.09343700	-1.51825300	0.00000000
C	-2.28839700	-0.09298200	0.00000000
C	-1.41806000	0.96210900	0.00000000
C	0.00000000	0.96288500	0.00000000
C	0.77859100	2.08441600	0.00000000
C	2.19977400	1.76044000	0.00000000
C	2.43909400	0.42386100	0.00000000

$C_6B_4H_{12}$ (k=2) Isomer I

H	-3.42634900	-1.98938200	0.00000000
H	-1.08492900	-3.45584600	0.00000000
H	1.31780800	-2.41894400	0.00000000
H	-3.54661600	0.62219500	0.00000000
H	-1.85251900	2.18076700	0.00000000
H	3.07624600	-0.79576400	0.00000000
H	3.59808200	1.67615100	0.00000000
H	1.20365600	3.36503200	0.00000000
B	-2.43951300	-1.32883800	0.00000000
B	-0.92621100	-2.27913600	0.00000000
H	-1.66788800	-1.78496300	0.97030800
H	-1.66788800	-1.78496300	-0.97030800
B	0.00000000	0.92025300	0.00000000
B	1.29294800	2.18216700	0.00000000
H	0.48823000	1.74622700	0.95008000
H	0.48823000	1.74622700	-0.95008000
C	-2.53366200	0.22430500	0.00000000
C	-1.53978500	1.13633600	0.00000000
C	0.51137200	-1.68548300	0.00000000
C	0.90310800	-0.38629000	0.00000000
C	2.31609500	-0.02057500	0.00000000
C	2.58250700	1.30187800	0.00000000

$C_6B_4H_{12}$ (k=2) Isomer II

H	-3.14866900	1.94872900	0.00000000
H	-3.37607200	-0.32441100	0.00000000
H	-1.78892000	-1.99714200	0.00000000
H	-1.25755100	3.27814600	0.00000000
H	0.97071600	2.66735400	0.00000000
H	0.38740300	-3.31509400	0.00000000
H	3.49393800	-2.52882700	0.00000000
H	3.50775500	0.69554800	0.00000000
B	0.82594300	0.41082400	0.00000000
B	2.59132400	-0.06354200	0.00000000
H	1.82764400	0.33296300	0.95586200
H	1.82764400	0.33296300	-0.95586200
B	2.58629100	-1.76224700	0.00000000
B	0.85604000	-2.22070100	0.00000000
H	1.77837100	-2.20279400	0.95145600
H	1.77837100	-2.20279400	-0.95145600
C	0.00000000	-0.90662600	0.00000000
C	-1.36318200	-0.99618100	0.00000000
C	-2.35337200	0.03658800	0.00000000
C	-2.21770700	1.39242100	0.00000000
C	-1.04390000	2.21320300	0.00000000
C	0.26172500	1.84287900	0.00000000

$C_6B_4H_{12}$ (k=2) Isomer III

H	-3.18348100	2.07253200	0.00000000
H	-3.37321900	-0.20517600	0.00000000
H	-1.82476500	-1.85170900	0.00000000
H	-1.04105300	3.59881600	0.00000000
H	1.57758100	2.51502800	0.00000000
H	0.19720300	-3.35862300	0.00000000
H	3.42440400	-2.43837600	0.00000000
H	3.09441700	0.44973500	0.00000000
B	-0.95591500	2.41460100	0.00000000
B	0.68389600	1.73327400	0.00000000
H	-0.13611600	2.08782800	0.97185800
H	-0.13611600	2.08782800	-0.97185800
B	2.42714700	-1.79815300	0.00000000
B	0.72579600	-2.29737600	0.00000000
H	1.63906400	-2.32655800	0.94979200
H	1.63906400	-2.32655800	-0.94979200
C	0.98240800	0.20313400	0.00000000
C	2.26794400	-0.25014200	0.00000000
C	0.00000000	-0.90099500	0.00000000
C	-1.36246300	-0.86935400	0.00000000
C	-2.36084200	0.18944100	0.00000000
C	-2.24064700	1.53349900	0.00000000

$C_6B_4H_{12}$ (k=2) Isomer IV

H	-3.22805500	-2.29037000	0.00000000
H	-0.67485700	-3.59341600	0.00000000
H	1.54465200	-2.23833600	0.00000000
H	-3.42222900	0.31876000	0.00000000
H	-1.83074900	1.93762000	0.00000000
H	3.37886300	-0.49512600	0.00000000
H	3.01313800	2.84478300	0.00000000
H	0.12655800	3.00636100	0.00000000
B	-2.26386200	-1.59711600	0.00000000
B	-0.68807400	-2.40569500	0.00000000
H	-1.48449000	-2.04524600	0.97190100
H	-1.48449000	-2.04524600	-0.97190100
B	2.22027400	1.96421000	0.00000000
B	2.42580400	0.20927700	0.00000000
H	2.61611200	1.09878500	0.94995100
H	2.61611200	1.09878500	-0.94995100
C	-2.40140500	-0.05278800	0.00000000
C	-1.46339300	0.91444400	0.00000000
C	0.00000000	0.88388100	0.00000000
C	0.67335700	2.07144200	0.00000000
C	0.92695600	-0.28262500	0.00000000
C	0.65760600	-1.60947700	0.00000000

$C_6B_4H_{12}$ (k=2) Isomer V

H	-3.15445900	2.10010500	0.00000000
H	-3.34664800	-0.14555900	0.00000000
H	-1.84753000	-1.82655100	0.00000000
H	-1.13226900	3.69416100	0.00000000
H	1.55013100	2.80213200	0.00000000
H	0.10911800	-3.09706500	0.00000000
H	2.63501000	-3.01448600	0.00000000
H	3.69605800	-0.28764200	0.00000000
B	-0.96350000	2.51795700	0.00000000
B	0.72503200	1.94625100	0.00000000
H	-0.10978900	2.21696100	0.96859600
H	-0.10978900	2.21696100	-0.96859600
B	1.05062400	0.30010800	0.00000000
B	2.56883800	-0.65612200	0.00000000
H	1.94783100	0.06232600	0.94586400
H	1.94783100	0.06232600	-0.94586400
C	2.02977500	-2.11714800	0.00000000
C	0.67930400	-2.17260000	0.00000000
C	0.00000000	-0.88667300	0.00000000
C	-1.36376600	-0.85134400	0.00000000
C	-2.32613200	0.22825800	0.00000000
C	-2.20092400	1.57873200	0.00000000

$C_6B_4H_{12}$ (k=2) Isomer VI

H	-2.89335600	2.57113000	0.00000000
H	-3.61428400	0.42252800	0.00000000
H	-2.38477200	-1.83239700	0.00000000
H	-0.59224900	3.65961700	0.00000000
H	1.86072900	2.39804900	0.00000000
H	0.47551100	-3.40556900	0.00000000
H	2.95469600	-2.69369200	0.00000000
H	3.25409300	-0.07788700	0.00000000
B	-0.66599200	2.47319800	0.00000000
B	0.93566800	1.65161200	0.00000000
H	0.10009900	2.05107700	0.96878700
H	0.10009900	2.05107700	-0.96878700
B	-1.76459300	-0.81816000	0.00000000
B	0.00000000	-1.02960300	0.00000000
H	-0.87207000	-0.92446400	0.97403300
H	-0.87207000	-0.92446400	-0.97403300
C	1.09756700	0.12347900	0.00000000
C	2.27467200	-0.54432400	0.00000000
C	2.10884300	-2.01629300	0.00000000
C	0.81222500	-2.37894700	0.00000000
C	-2.53246100	0.54575000	0.00000000
C	-2.10115200	1.82362700	0.00000000

$C_6B_4H_{12}$ (k=2) Isomer VII

H	-3.26440300	-1.85304000	0.00000000
H	-1.52351100	-3.25984300	0.00000000
H	1.03363400	-2.93299000	0.00000000
H	-3.48043000	0.37454000	0.00000000
H	-2.00419000	2.08878000	0.00000000
H	3.37007600	-0.56210100	0.00000000
H	3.60511100	1.90537000	0.00000000
H	1.20587700	3.45513500	0.00000000
B	0.30702700	-1.99038600	0.00000000
B	1.00129500	-0.35385500	0.00000000
H	0.70082100	-1.18742400	0.97002000
H	0.70082100	-1.18742400	-0.97002000
B	0.00000000	1.01582500	0.00000000
B	1.28454100	2.26794700	0.00000000
H	0.46525800	1.85462400	0.94720500
H	0.46525800	1.85462400	-0.94720500
C	-1.54305800	1.10314200	0.00000000
C	-2.43347700	0.08183600	0.00000000
C	-2.29166600	-1.36796100	0.00000000
C	-1.23194200	-2.21261800	0.00000000
C	2.50023100	0.08859900	0.00000000
C	2.62680700	1.43235200	0.00000000

$C_6B_4H_{12}$ (k=2) Isomer VIII

H	-3.06405300	-2.12788100	0.00000000
H	-1.19823300	-3.43584300	0.00000000
H	1.33637600	-2.79542500	0.00000000
H	-3.35058400	0.12211200	0.00000000
H	-1.92671400	1.86912200	0.00000000
H	3.67945800	-0.18115100	0.00000000
H	2.94616200	3.02076900	0.00000000
H	0.09936000	3.07637500	0.00000000
B	0.49150200	-1.96010000	0.00000000
B	1.02549100	-0.26784400	0.00000000
H	0.82083100	-1.11047500	0.97112100
H	0.82083100	-1.11047500	-0.97112100
B	2.21490700	2.08449000	0.00000000
B	2.61689000	0.34816500	0.00000000
H	2.68866600	1.24258100	0.94891400
H	2.68866600	1.24258100	-0.94891400
C	0.65480500	2.14183900	0.00000000
C	0.00000000	0.94757700	0.00000000
C	-1.44610100	0.89356800	0.00000000
C	-2.30256600	-0.15813600	0.00000000
C	-2.11584400	-1.59698300	0.00000000
C	-1.00441200	-2.36717200	0.00000000

$C_6B_4H_{12}$ (k=2) Isomer IX

H	-3.25808300	2.45866600	0.00000000
H	-3.59908400	-0.28153100	0.00000000
H	-1.75462400	-2.02729900	0.00000000
H	-0.58306100	3.78961700	0.00000000
H	1.81684700	2.34432200	0.00000000
H	0.37829200	-3.20109300	0.00000000
H	2.95033400	-2.55583000	0.00000000
H	3.14237300	0.12222400	0.00000000
B	-2.23459800	1.85495600	0.00000000
B	-2.46951200	0.08768600	0.00000000
H	-2.33096700	0.97168900	0.96755500
H	-2.33096700	0.97168900	-0.96755500
B	-0.71111500	2.60770500	0.00000000
B	0.82413600	1.69035200	0.00000000
H	0.02326800	2.16074600	0.96817200
H	0.02326800	2.16074600	-0.96817200
C	0.97407800	0.15456400	0.00000000
C	2.21023600	-0.42397300	0.00000000
C	2.11121400	-1.87693800	0.00000000
C	0.80327300	-2.20917200	0.00000000
C	0.00000000	-0.98138700	0.00000000
C	-1.35249200	-1.01600100	0.00000000

$C_6B_4H_{12}$ (k=2) Isomer X

H	-3.36078200	-2.09131500	0.00000000
H	-1.14986800	-3.71472700	0.00000000
H	1.66717100	-2.70282700	0.00000000
H	-3.40202100	0.44441500	0.00000000
H	-1.80626100	2.05154200	0.00000000
H	3.46316700	0.04116200	0.00000000
H	2.95267100	2.57223300	0.00000000
H	0.37632200	3.08127600	0.00000000
B	-2.33233800	-1.49546900	0.00000000
B	-0.88662800	-2.55557500	0.00000000
H	-1.56179000	-2.01579500	0.96698800
H	-1.56179000	-2.01579500	-0.96698800
B	0.71541600	-1.98981600	0.00000000
B	1.06816000	-0.25527500	0.00000000
H	0.91605100	-1.12478500	0.97097000
H	0.91605100	-1.12478500	-0.97097000
C	-2.38701800	0.05612500	0.00000000
C	-1.43816700	1.02631600	0.00000000
C	0.00000000	0.94935500	0.00000000
C	0.76836300	2.07049100	0.00000000
C	2.21021300	1.78340200	0.00000000
C	2.46794900	0.46099100	0.00000000

$C_4B_6H_{14}$ (k=3) Isomer I

H	-3.48744700	-1.99421700	0.00000000
H	-1.15101800	-3.48596200	0.00000000
H	1.24414800	-2.47780500	0.00000000
H	-3.56312200	0.62204800	0.00000000
H	-1.84475900	2.14803700	0.00000000
H	3.25039600	-1.03606100	0.00000000
H	3.78793600	2.13063700	0.00000000
H	0.83769000	3.41658000	0.00000000
B	-2.48970800	-1.35047500	0.00000000
B	-0.98930400	-2.30940100	0.00000000
H	-1.72597400	-1.81925800	0.97099300
H	-1.72597400	-1.81925800	-0.97099300
B	0.00000000	0.87351000	0.00000000
B	1.16884100	2.27439900	0.00000000
H	0.49045800	1.71893600	0.95359100
H	0.49045800	1.71893600	-0.95359100
B	2.72839500	1.59413200	0.00000000
B	2.44279500	-0.16227100	0.00000000
H	2.81648100	0.68545700	0.95112400
H	2.81648100	0.68545700	-0.95112400
C	-2.55716800	0.20647100	0.00000000
C	-1.54850400	1.09867000	0.00000000
C	0.46004800	-1.72052500	0.00000000
C	0.88881500	-0.43345000	0.00000000

$C_4B_6H_{14}$ (k=3) Isomer II

H	-2.95985700	-2.91157100	0.00000000
H	-0.26827900	-3.63431100	0.00000000
H	1.68991900	-2.07331600	0.00000000
H	-3.84383600	-0.04883500	0.00000000
H	-2.08709700	2.07355300	0.00000000
H	3.42856600	-0.29977000	0.00000000
H	2.96505600	3.02434100	0.00000000
H	0.07111200	3.09149700	0.00000000
B	-2.20090600	-1.99729200	0.00000000
B	-0.48474100	-2.46529500	0.00000000
H	-1.35305400	-2.22700300	0.96779300
H	-1.35305400	-2.22700300	-0.96779300
B	-2.70025100	-0.37161300	0.00000000
B	-1.56961900	1.00414500	0.00000000
H	-2.12641200	0.28387500	0.96770600
H	-2.12641200	0.28387500	-0.96770600
B	2.20436500	2.11580300	0.00000000
B	2.45295300	0.37279700	0.00000000
H	2.62208900	1.27657800	0.94942600
H	2.62208900	1.27657800	-0.94942600
C	0.00000000	0.96877900	0.00000000
C	0.64147900	2.17096600	0.00000000
C	0.96570400	-0.16354000	0.00000000
C	0.76117900	-1.50640700	0.00000000

$C_4B_6H_{14}$ (k=3) Isomer III

H	-3.01117700	2.54092600	0.00000000
H	-3.67512000	0.36119000	0.00000000
H	-2.37560400	-1.84405100	0.00000000
H	-0.70584200	3.65483800	0.00000000
H	1.73417600	2.44931900	0.00000000
H	0.39598100	-3.66147400	0.00000000
H	3.48833000	-2.55662600	0.00000000
H	3.19708900	0.27833700	0.00000000
B	-0.77730700	2.46882800	0.00000000
B	0.83104100	1.67647400	0.00000000
H	-0.00235600	2.03947000	0.96893000
H	-0.00235600	2.03947000	-0.96893000
B	-1.77740100	-0.81710300	0.00000000
B	0.00000000	-1.02876400	0.00000000
H	-0.87992500	-0.91024600	0.96922700
H	-0.87992500	-0.91024600	-0.96922700
B	2.46652800	-1.95028900	0.00000000
B	0.79728500	-2.54459000	0.00000000
H	1.70534000	-2.51881000	0.94776300
H	1.70534000	-2.51881000	-0.94776300
C	-2.20524100	1.80862300	0.00000000
C	-2.59793600	0.52072900	0.00000000
C	1.07471500	0.13701400	0.00000000
C	2.32934800	-0.37737700	0.00000000

$C_4B_6H_{14}$ (k=3) Isomer IV

H	-3.38157100	2.36615000	0.00000000
H	-3.60254400	-0.31502300	0.00000000
H	-1.76619100	-2.05323400	0.00000000
H	-0.85868100	3.91652200	0.00000000
H	1.66821000	2.69164800	0.00000000
H	0.25716600	-3.19037700	0.00000000
H	2.77425400	-2.95522600	0.00000000
H	3.65678100	-0.16442200	0.00000000
B	-2.31752500	1.83727000	0.00000000
B	-2.47367600	0.05568800	0.00000000
H	-2.33053100	0.95676800	0.96633700
H	-2.33053100	0.95676800	-0.96633700
B	-0.86613500	2.72736500	0.00000000
B	0.73681500	1.94961800	0.00000000
H	-0.06786900	2.32939000	0.96516800
H	-0.06786900	2.32939000	-0.96516800
B	0.98523400	0.26988200	0.00000000
B	2.55615200	-0.60561000	0.00000000
H	1.88357100	0.07685200	0.94511200
H	1.88357100	0.07685200	-0.94511200
C	-1.35628500	-1.04222600	0.00000000
C	0.00000000	-0.99038800	0.00000000
C	0.77259400	-2.23451700	0.00000000
C	2.11334100	-2.09839000	0.00000000

$C_4B_6H_{14}$ (k=3) Isomer V

H	-3.30719000	1.96821000	0.00000000
H	-3.38322700	-0.28580100	0.00000000
H	-1.82165700	-1.88416300	0.00000000
H	-1.34941400	3.65491600	0.00000000
H	1.36239700	2.86872400	0.00000000
H	0.12117500	-3.33087500	0.00000000
H	3.29985100	-2.90016800	0.00000000
H	3.64993300	0.29830300	0.00000000
B	-1.13228800	2.48688600	0.00000000
B	0.57166700	1.98064600	0.00000000
H	-0.26738600	2.21853400	0.96914500
H	-0.26738600	2.21853400	-0.96914500
B	0.98265200	0.34070900	0.00000000
B	2.65632900	-0.35402500	0.00000000
H	1.91768500	0.16597000	0.94819400
H	1.91768500	0.16597000	-0.94819400
B	2.47470400	-2.04585500	0.00000000
B	0.70954100	-2.29630600	0.00000000
H	1.63520600	-2.39196800	0.95170100
H	1.63520600	-2.39196800	-0.95170100
C	-2.32967900	1.49322500	0.00000000
C	-2.38393500	0.14206100	0.00000000
C	-1.36237000	-0.89747100	0.00000000
C	0.00000000	-0.89356500	0.00000000

$C_4B_6H_{14}$ (k=3) Isomer VI

H	-2.97967300	2.58417800	0.00000000
H	-3.63098400	0.43083000	0.00000000
H	-2.40502300	-1.79545500	0.00000000
H	-0.76608900	3.77559100	0.00000000
H	1.72446800	2.74601400	0.00000000
H	0.21961300	-3.38835800	0.00000000
H	2.69595900	-3.17050600	0.00000000
H	3.75691000	-0.52437700	0.00000000
B	-0.76250800	2.58698800	0.00000000
B	0.89295000	1.89604000	0.00000000
H	0.04951500	2.17176000	0.96544000
H	0.04951500	2.17176000	-0.96544000
B	-1.77121500	-0.78796400	0.00000000
B	0.00000000	-1.01583100	0.00000000
H	-0.87943800	-0.86625100	0.96881600
H	-0.87943800	-0.86625100	-0.96881600
B	1.17461900	0.22856700	0.00000000
B	2.60520800	-0.82155800	0.00000000
H	2.04670900	-0.03096800	0.94309700
H	2.04670900	-0.03096800	-0.94309700
C	-2.15987000	1.86682400	0.00000000
C	-2.55133800	0.57506400	0.00000000
C	2.04809400	-2.29823100	0.00000000
C	0.70577700	-2.41669300	0.00000000

$C_4B_6H_{14}$ (k=3) Isomer VII

H	-3.58810400	-1.84411700	0.00000000
H	-1.55850100	-3.57718700	0.00000000
H	1.34048300	-2.92288500	0.00000000
H	-3.54572300	0.68197900	0.00000000
H	-1.88344400	2.22938000	0.00000000
H	3.41342700	-0.53246100	0.00000000
H	3.60611000	1.94260700	0.00000000
H	1.16325300	3.43318600	0.00000000
B	-2.53235500	-1.29838100	0.00000000
B	-1.16540700	-2.45569300	0.00000000
H	-1.78843900	-1.83091900	0.96550800
H	-1.78843900	-1.83091900	-0.96550800
B	0.49620400	-2.08305100	0.00000000
B	1.04492100	-0.38720900	0.00000000
H	0.78612400	-1.24975400	0.96612500
H	0.78612400	-1.24975400	-0.96612500
B	0.00000000	0.98222800	0.00000000
B	1.27613400	2.24924900	0.00000000
H	0.46863300	1.80418700	0.94764900
H	0.46863300	1.80418700	-0.94764900
C	-2.53655200	0.27185400	0.00000000
C	-1.54932700	1.19103100	0.00000000
C	2.53437100	0.10611400	0.00000000
C	2.63857100	1.44879300	0.00000000

$C_4B_6H_{14}$ (k=3) Isomer VIII

H	-3.39552900	-2.15214800	0.00000000
H	-1.14861700	-3.78504900	0.00000000
H	1.63391800	-2.70556800	0.00000000
H	-3.41221800	0.37174700	0.00000000
H	-1.85103300	1.98002500	0.00000000
H	3.70411300	-0.14640800	0.00000000
H	2.93261200	3.05772000	0.00000000
H	0.10073700	3.06206200	0.00000000
B	-2.35656100	-1.57419400	0.00000000
B	-0.91204800	-2.62005200	0.00000000
H	-1.60921500	-2.10487600	0.96782100
H	-1.60921500	-2.10487600	-0.96782100
B	0.67416400	-2.00397900	0.00000000
B	1.05338400	-0.26249000	0.00000000
H	0.86696500	-1.13361900	0.96631500
H	0.86696500	-1.13361900	-0.96631500
B	2.22959600	2.10013300	0.00000000
B	2.64237100	0.38382800	0.00000000
H	2.72522200	1.28385600	0.94760200
H	2.72522200	1.28385600	-0.94760200
C	-2.39564100	-0.01427400	0.00000000
C	-1.46255100	0.96258600	0.00000000
C	0.00000000	0.94037200	0.00000000
C	0.66078100	2.12975900	0.00000000

$C_4B_6H_{14}$ (k=3) Isomer IX

H	-3.17437600	-2.73476100	0.00000000
H	-0.69733700	-3.87288700	0.00000000
H	1.85741700	-2.47063400	0.00000000
H	-3.81071600	0.08694100	0.00000000
H	-2.08816100	2.19744200	0.00000000
H	3.48536400	0.20987600	0.00000000
H	2.94607600	2.73810700	0.00000000
H	0.35471400	3.20341600	0.00000000
B	-2.29714700	-1.93149100	0.00000000
B	-0.67288000	-2.68382900	0.00000000
H	-1.45843300	-2.26718800	0.96290900
H	-1.45843300	-2.26718800	-0.96290900
B	-2.67303600	-0.26048600	0.00000000
B	-1.54280800	1.14031300	0.00000000
H	-2.09178800	0.37569400	0.96599300
H	-2.09178800	0.37569400	-0.96599300
B	0.83245100	-1.86701600	0.00000000
B	1.08260900	-0.10602200	0.00000000
H	0.91455700	-0.99027500	0.96963500
H	0.91455700	-0.99027500	-0.96963500
C	0.00000000	1.07069700	0.00000000
C	0.75584500	2.19538300	0.00000000
C	2.21654800	1.93713200	0.00000000
C	2.48634100	0.62157000	0.00000000

$C_4B_6H_{14}$ (k=3) Isomer X

H	-3.31619800	-1.88050500	0.00000000
H	-1.57754100	-3.30285400	0.00000000
H	0.97102400	-2.97770100	0.00000000
H	-3.50072400	0.35630600	0.00000000
H	-1.99793400	2.04403800	0.00000000
H	3.60559500	-0.76137700	0.00000000
H	3.75523600	2.38033400	0.00000000
H	0.81242600	3.52206800	0.00000000
B	0.25354600	-2.02861000	0.00000000
B	0.99358500	-0.41069300	0.00000000
H	0.67980400	-1.21934400	0.96777100
H	0.67980400	-1.21934400	-0.96777100
B	0.00000000	0.96623600	0.00000000
B	1.14361500	2.37694000	0.00000000
H	0.45526100	1.81987300	0.95145000
H	0.45526100	1.81987300	-0.95145000
B	2.74184500	1.75565700	0.00000000
B	2.66055000	-0.03667300	0.00000000
H	2.90467100	0.84475200	0.95012900
H	2.90467100	0.84475200	-0.95012900
C	-1.55150700	1.05094300	0.00000000
C	-2.45845500	0.04711000	0.00000000
C	-2.33802000	-1.40637300	0.00000000
C	-1.28486100	-2.25587300	0.00000000

$C_2B_8H_{16}$ (k=4) Isomer I

H	-3.23006700	-2.66795500	0.00000000
H	-0.69788800	-3.59260500	0.00000000
H	1.45198200	-2.30613300	0.00000000
H	-4.02966100	0.19246000	0.00000000
H	-2.15870100	2.27353200	0.00000000
H	3.33734400	-0.80866100	0.00000000
H	3.74524400	2.37667700	0.00000000
H	0.74670100	3.53044300	0.00000000
B	-2.43326600	-1.78671000	0.00000000
B	-0.75911300	-2.40573100	0.00000000
H	-1.58745600	-2.03558000	0.96638400
H	-1.58745600	-2.03558000	-0.96638400
B	-2.89003800	-0.14602700	0.00000000
B	-1.69834100	1.17537900	0.00000000
H	-2.27892000	0.50643800	0.96512800
H	-2.27892000	0.50643800	-0.96512800
B	0.00000000	0.97277300	0.00000000
B	1.12147300	2.40287800	0.00000000
H	0.42415700	1.81607100	0.94689500
H	0.42415700	1.81607100	-0.94689500
B	2.71087900	1.79319400	0.00000000
B	2.49291700	0.02936300	0.00000000
H	2.83326100	0.89556500	0.95143800
H	2.83326100	0.89556500	-0.95143800
C	0.60540600	-1.61840100	0.00000000
C	0.94966100	-0.30465600	0.00000000

$C_2B_8H_{16}$ (k=4) Isomer II

H	-3.20461000	-2.81558500	0.00000000
H	-0.68213800	-3.93342100	0.00000000
H	1.82135700	-2.48183100	0.00000000
H	-3.81318000	0.01673100	0.00000000
H	-2.13975300	2.10206200	0.00000000
H	3.73195800	0.05466700	0.00000000
H	2.89952800	3.24008000	0.00000000
H	0.06177700	3.18371900	0.00000000
B	-2.32107200	-2.01911800	0.00000000
B	-0.68880600	-2.74376800	0.00000000
H	-1.49174700	-2.35806800	0.96352900
H	-1.49174700	-2.35806800	-0.96352900
B	-2.68046500	-0.34457500	0.00000000
B	-1.56577200	1.06076100	0.00000000
H	-2.07925700	0.28732100	0.96501000
H	-2.07925700	0.28732100	-0.96501000
B	0.79352100	-1.88389600	0.00000000
B	1.07759400	-0.11212100	0.00000000
H	0.87429000	-1.00106400	0.96411100
H	0.87429000	-1.00106400	-0.96411100
B	2.21810700	2.26687900	0.00000000
B	2.65818800	0.55966100	0.00000000
H	2.72317600	1.46938600	0.94702000
H	2.72317600	1.46938600	-0.94702000
C	0.00000000	1.05984000	0.00000000
C	0.63594300	2.26004500	0.00000000

$C_2B_8H_{16}$ (k=4) Isomer III

H	-3.16452600	2.84456200	0.00000000
H	-3.93805600	0.29216000	0.00000000
H	-2.30201300	-2.09892300	0.00000000
H	-0.52063900	3.95305200	0.00000000
H	1.85107400	2.66253500	0.00000000
H	0.35579100	-3.44619100	0.00000000
H	2.81972000	-3.10311400	0.00000000
H	3.73408600	-0.39919200	0.00000000
B	-2.23286800	2.10556600	0.00000000
B	-2.75324500	0.39111300	0.00000000
H	-2.41046000	1.22540100	0.96152100
H	-2.41046000	1.22540100	-0.96152100
B	-0.64981100	2.77073600	0.00000000
B	0.92433200	1.91574800	0.00000000
H	0.09770900	2.27884600	0.96216300
H	0.09770900	2.27884600	-0.96216300
B	-1.78780200	-1.02466900	0.00000000
B	0.00000000	-1.09556000	0.00000000
H	-0.90610900	-1.00015500	0.96442800
H	-0.90610900	-1.00015500	-0.96442800
B	1.11589800	0.22305400	0.00000000
B	2.60200200	-0.76306500	0.00000000
H	1.99284100	-0.00979900	0.94287700
H	1.99284100	-0.00979900	-0.94287700
C	2.12702500	-2.26629300	0.00000000
C	0.79365400	-2.45172300	0.00000000

$C_2B_8H_{16}$ (k=4) Isomer IV

H	3.64815000	1.86011300	0.00000000
H	1.62218300	3.61798500	0.00000000
H	-1.26486600	2.97555400	0.00000000
H	3.56074000	-0.66768300	0.00000000
H	1.87566300	-2.18377100	0.00000000
H	-3.64736000	0.73744000	0.00000000
H	-3.75508200	-2.41623800	0.00000000
H	-0.78531500	-3.48591500	0.00000000
B	2.58418800	1.33073700	0.00000000
B	1.22882100	2.49638300	0.00000000
H	1.84831400	1.87395700	0.96605400
H	1.84831400	1.87395700	-0.96605400
B	-0.43409100	2.12307100	0.00000000
B	-1.03534100	0.44096300	0.00000000
H	-0.76055300	1.28137700	0.96294400
H	-0.76055300	1.28137700	-0.96294400
B	0.00000000	-0.93444100	0.00000000
B	-1.14589200	-2.35028400	0.00000000
H	-0.47192800	-1.76548900	0.95096700
H	-0.47192800	-1.76548900	-0.95096700
B	-2.75759500	-1.76717100	0.00000000
B	-2.69833300	0.01795900	0.00000000
H	-2.94372200	-0.86271800	0.94973100
H	-2.94372200	-0.86271800	-0.94973100
C	2.55906300	-0.23933900	0.00000000
C	1.55641500	-1.14029900	0.00000000

$C_2B_8H_{16}$ (k=4) Isomer V

H	-3.16196800	2.46374400	0.00000000
H	-3.70296900	0.27466600	0.00000000
H	-2.37559700	-1.87348200	0.00000000
H	-0.99284300	3.75160100	0.00000000
H	1.52947200	2.82092900	0.00000000
H	0.27384400	-3.65063300	0.00000000
H	3.36563300	-3.04764700	0.00000000
H	3.76055500	0.07407700	0.00000000
B	-0.94301200	2.56431900	0.00000000
B	0.73399300	1.93655900	0.00000000
H	-0.11769600	2.17336300	0.96555300
H	-0.11769600	2.17336300	-0.96555300
B	-1.78109700	-0.84332500	0.00000000
B	0.00000000	-1.02654800	0.00000000
H	-0.86772500	-0.89726700	0.96599900
H	-0.86772500	-0.89726700	-0.96599900
B	1.10941200	0.27844200	0.00000000
B	2.72431700	-0.51367400	0.00000000
H	2.01368500	0.07821600	0.94603200
H	2.01368500	0.07821600	-0.94603200
B	2.51183700	-2.21811200	0.00000000
B	0.75455500	-2.56131300	0.00000000
H	1.67317400	-2.59157000	0.95019300
H	1.67317400	-2.59157000	-0.95019300
C	-2.30894300	1.78636700	0.00000000
C	-2.63256200	0.47688600	0.00000000

$B_{10}H_{18}$ (k=5)

H	3.46622500	2.58460500	0.00000000
H	1.10734900	3.85080300	0.00000000
H	-1.54187900	2.72761600	0.00000000
H	4.00036100	-0.24075000	0.00000000
H	2.25063000	-2.28100500	0.00000000
H	-3.69586600	0.50611300	0.00000000
H	-3.72423500	-2.64979600	0.00000000
H	-0.73704700	-3.64092900	0.00000000
B	2.55346800	1.82255000	0.00000000
B	0.97657200	2.66885700	0.00000000
H	1.72687300	2.17446600	0.96178300
H	1.72687300	2.17446600	-0.96178300
B	2.87272000	0.13652800	0.00000000
B	1.70748200	-1.22174800	0.00000000
H	2.22709600	-0.48675000	0.96180200
H	2.22709600	-0.48675000	-0.96180200
B	-0.60247400	1.99714400	0.00000000
B	-1.07347500	0.26497200	0.00000000
H	-0.77787300	1.11267100	0.96074500
H	-0.77787300	1.11267100	-0.96074500
B	0.00000000	-1.08826900	0.00000000
B	-1.11591700	-2.51190700	0.00000000
H	-0.40254200	-1.91950300	0.94579400
H	-0.40254200	-1.91950300	-0.94579400
B	-2.74506700	-1.97305600	0.00000000
B	-2.72787300	-0.18730500	0.00000000
H	-2.94991000	-1.07863000	0.94984200
H	-2.94991000	-1.07863000	-0.94984200
